# A refined compilation of implementation strategies: results from the Expert Recommendations for Implementing Change (ERIC) project

**DOI:** 10.1186/s13012-015-0209-1

**Published:** 2015-02-12

**Authors:** Byron J Powell, Thomas J Waltz, Matthew J Chinman, Laura J Damschroder, Jeffrey L Smith, Monica M Matthieu, Enola K Proctor, JoAnn E Kirchner

**Affiliations:** Center for Mental Health Policy and Services Research, Department of Psychiatry, Perelman School of Medicine, University of Pennsylvania, 3535 Market Street, 3rd Floor, Philadelphia, PA 19104 USA; Department of Psychology, Eastern Michigan University, Ypsilanti, MI USA; VISN 4 MIRECC, Pittsburgh, PA USA; RAND Corporation, Pittsburgh, PA USA; HSR&D Center for Clinical Management Research, VA Ann Arbor Healthcare System, Ann Arbor, MI USA; Central Arkansas Veterans Healthcare System, HSR&D and Mental Health Quality Enhancement Research Initiative (QUERI), Department of Veterans Affairs Medical Center, Little Rock, AR USA; School of Social Work, College for Public Health & Social Justice, Saint Louis University, St. Louis, MO USA; Brown School, Washington University in St. Louis, St. Louis, MO USA; Department of Psychiatry, College of Medicine, University of Arkansas for Medical Sciences, Little Rock, AR USA

**Keywords:** Implementation research, Implementation strategies, Knowledge translation strategies, Mental health, US Department of Veterans Affairs

## Abstract

**Background:**

Identifying, developing, and testing implementation strategies are important goals of implementation science. However, these efforts have been complicated by the use of inconsistent language and inadequate descriptions of implementation strategies in the literature. The Expert Recommendations for Implementing Change (ERIC) study aimed to refine a published compilation of implementation strategy terms and definitions by systematically gathering input from a wide range of stakeholders with expertise in implementation science and clinical practice.

**Methods:**

Purposive sampling was used to recruit a panel of experts in implementation and clinical practice who engaged in three rounds of a modified Delphi process to generate consensus on implementation strategies and definitions. The first and second rounds involved Web-based surveys soliciting comments on implementation strategy terms and definitions. After each round, iterative refinements were made based upon participant feedback. The third round involved a live polling and consensus process via a Web-based platform and conference call.

**Results:**

Participants identified substantial concerns with 31% of the terms and/or definitions and suggested five additional strategies. Seventy-five percent of definitions from the originally published compilation of strategies were retained after voting. Ultimately, the expert panel reached consensus on a final compilation of 73 implementation strategies.

**Conclusions:**

This research advances the field by improving the conceptual clarity, relevance, and comprehensiveness of implementation strategies that can be used in isolation or combination in implementation research and practice. Future phases of ERIC will focus on developing conceptually distinct categories of strategies as well as ratings for each strategy’s importance and feasibility. Next, the expert panel will recommend multifaceted strategies for hypothetical yet real-world scenarios that vary by sites’ endorsement of evidence-based programs and practices and the strength of contextual supports that surround the effort.

**Electronic supplementary material:**

The online version of this article (doi:10.1186/s13012-015-0209-1) contains supplementary material, which is available to authorized users.

## Background

Research focusing on implementation strategies, defined as “methods or techniques used to enhance the adoption, implementation, and sustainability of a clinical program or practice” [1], has been prioritized in order to bridge the quality chasm in health and mental health services [2-5].^a^ However, efforts to identify, develop, and test implementation strategies have been complicated by a lack of conceptual clarity [1,6-9]. This lack of conceptual clarity manifests in two primary ways. First, terms and definitions for implementation strategies are inconsistent [7,10]. Idiosyncratic use of implementation strategy terms involve homonymy (*i.e.*, same term has multiple meanings), synonymy (*i.e.*, different terms have the same meanings), and instability (*i.e.*, terms shift unpredictably over time) [11]. Implementation scientists have responded by calling for efforts to clarify terminology and use it consistently [1,5-7,12]. Second, published descriptions of implementation strategies too often do not include sufficient detail to enable either scientific or real-world replication [1,6], leading some to suggest guidelines for specifying and reporting implementation strategies [1,6,13,14]. Taken together, these two deficiencies complicate the acquisition and interpretation of knowledge, preclude research syntheses such as systematic reviews and meta-analyses, limit replication in both research and practice, and ultimately stymie the translation and application of empirical studies that could inform implementation processes [1,6,9].

A number of taxonomies of implementation strategies have been developed, in part, to address these shortcomings pertaining to the published literature, *e.g.*, [10,15-18]. Powell *et al.* [10] reviewed 41 compilations and reviews of implementation strategies and summarized them according to their foci and disciplines/clinical specialties that they represented (this can be found in Table One of that publication). While they acknowledge that many of those compilations represent seminal contributions to the field, they also argue that most of the compilations were not necessarily intended to be consolidated “menus” of potential implementation strategies for a broad range of stakeholders in health and mental health. Powell *et al.* [10] note that many compilations and reviews:

are purposely narrow in scope, focusing on strategies with known evidence on effectiveness, *e.g.* [19-22]; specific medical conditions, fields of practice, or disciplines, *e.g.* [23-25]; strategies that were used in a specific setting or study, *e.g.* [26,27]; “exemplar” programs or strategies, *e.g.* [28,29]; one level of target such as consumers or practitioners, *e.g.* [30]; or one type of strategy such as educational or organizational strategies, *e.g.* [24,31]. The characteristics of some of these reviews and compilations may lead health care stakeholders to believe that there are relatively few strategies from which to choose. Additionally, many of these compilations do not provide definitions or provide definitions that do not adequately describe the specific actions that need to be taken by stakeholders.

In response to those limitations, Powell *et al.* [10] proposed a consolidated compilation of 68 discrete (as opposed to multifaceted) implementation strategies and definitions based upon a review of the health and mental health literatures. While the review was conducted by an interdisciplinary team of health services researchers, the development of the compilation was not informed by a wide-range of implementation and clinical experts, and the authors did not seek to generate consensus on the strategy terms and definitions beyond the study team [10]. This raises the question of whether the strategy terms and definitions identified would resonate with a broader array of researchers and implementers in real-world settings. The Expert Recommendations for Implementing Change (ERIC) study [9] builds upon the Powell *et al*. [10] review by generating expert consensus “on a common nomenclature for implementation strategy terms, definitions, and categories that can be used to guide implementation research and practice in mental health service settings” [9]. We pursued this aim by recruiting a panel of stakeholders with expertise in implementation science and clinical practice and engaging them in a three-round modified-Delphi process to refine Powell *et al.*’s [10] compilation of implementation strategies. While many other efforts to generate consensus have relied solely upon qualitative approaches, *e.g.*, [8,10,32], this study’s mixed methods approach provides more structure for the expert recommendation process and derives consensus quantitatively. We describe these processes below, and more details about our methodological approach have been published elsewhere [9].

## Methods

### Expert panel participants

We employed a purposive sampling procedure [33] that began with an initial list of implementation science experts generated by members of the study team. The team targeted a number of groups based upon their substantial expertise in implementation research, including members of the editorial board for the journal *Implementation Science*, implementation research coordinators for the VA Quality Enhancement Research Initiatives (QUERIs) [34], and faculty and fellows from the National Institute of Mental Health funded Implementation Research Institute [35]. Nominees were encouraged to identify peers with expertise in implementation science and clinical management related to implementing evidence-based programs and practices. Efforts were made to ensure a diverse sample by including VA and non-VA implementation experts and by attempting to obtain a balance between implementation and clinical expertise. Recruitment was limited to individuals residing in the four primary time zones of North America (*i.e.*, Eastern through Pacific) in order to minimize scheduling conflicts for the live Webinar (described below). Ultimately, we recruited a panel of 71 experts (see “Contributors” section for a full list of participants), each of whom participated in at least one of the three Delphi rounds (see Table 1). Ninety-seven percent of the experts were affiliated with academic or health-care institutions in the USA, and 3% were affiliated with Canadian universities. Ninety percent of participants had expertise in implementation science and practice, and 45% were also experts in clinical practice. Nearly two-thirds of participants had some affiliation with the VA, though most of those individuals also had academic appointments in social science or health-related schools or departments.

**Table 1:** **Composition of expert panel (** ***n*** **= 71)**

**Round**	**Participants**	**VA (%)**	**Female (%)**	**Type of expertise**		
				**Implementation (%)**	**Clinical (%)**	**Both (%)**
1	57	65	65	56	9	35
2	43	65	79	56	9	35
3	40	75	70	60	10	30
Total	71	66	65	55	10	35

Total represents the total number of unique experts participating in at least one round of the modified Delphi process.

### Modified Delphi process

The modified Delphi process [36] had three rounds. The first two rounds provided the opportunity for panel members to offer feedback on a list of strategies and definitions via two Web-based surveys. After each of the first two rounds, iterative refinements were made to the compilation based upon participant feedback. The third round involved a live, Web-based polling process to obtain consensus on the final compilation of strategies.

#### Round 1

Fifty-seven experts completed the Round 1 Web-based survey. Section one of the Round 1 survey listed terms and definitions from Powell *et al.*’s [10] published taxonomy of 68 strategies. Each “item” included a strategy term, its definition, a text box for participants to write in possible synonyms, and a text box for further comments, proposed definitions, or concerns regarding the strategy term or definition. Section 2 of the Round 1 survey asked panelists to propose strategy terms and definitions not included in Powell *et al.*’s [10] compilation. The full survey can be viewed in Additional file 1.

#### Round 2

Forty-three experts completed the Round 2 Web-based survey, which included the implementation strategy terms and definitions from Round 1 along with a summary of the panelists’ comments and suggestions regarding additional strategies. This included both a qualitative summary and, where possible, a quantitative characterization of participants’ Round 1 responses (*e.g.*, 72% of panelists made no comment). The core definitions from the original compilation [10] were separated from their accompanying “ancillary material” (additional details that may be helpful in understanding the nuances of the strategy). This allowed us to summarize and group the feedback from Round 1 according to whether the concerns panel members expressed pertained to the core definition, alternate definitions (proposed by participants in Round 1), or concerns or addendum to the ancillary material. The full Round 2 survey can be viewed in Additional file 2. Once again, participants could suggest additional strategies and make additional comments in response to the strategies, definitions, or feedback from Round 1. Panelists’ feedback from Round 2 was used to construct a final list of strategies and definitions for the consensus meeting in Round 3. Terms and definitions were considered “acceptable” to the expert panel and were not included in the Round 3 voting if no panelist suggested alternatives or expressed concerns about the core definition.

#### Round 3

Forty experts participated in Round 3 of the modified Delphi, which involved a live polling and consensus process conducted via a Web-based interactive discussion platform. Prior to the meeting, panelists were e-mailed a voting guide describing the voting process along with a ballot, allowing them to prepare responses in advance (the voting guide and ballot can be viewed in Additional files 3 and 4, respectively). During the consensus meeting, each implementation strategy term and core definition for which concerns were raised during Round 1 or 2 was presented along with the alternative definitions proposed from the earlier rounds. Terms with only one alternative definition were presented first, followed by those with multiple alternatives. This strategy was used so panelists could “warm up” by voting under the least complicated circumstances, with voting continuing with increasingly difficult scenarios and ending with voting on new terms proposed by panelists. The first stage of voting involved “approval voting”, in which panelists were given the option to vote for as many definitions (original and alternative) they thought acceptable. Approval voting is particularly useful for efficiently identifying the most acceptable choice [37], as it has been deemed the most “sincere and strategy proof” form of voting [38]. It promotes collaborative versus adversarial forms of decision making. Furthermore, it allowed us to determine whether the definitions from the original compilation [10] were acceptable even when alternative definitions may have been preferred. Approval ratings for existing definitions, when low, pointed to the need for improving definitional clarity. While no research literature could be found to support a supermajority cutoff, we drew upon supermajority benchmarks from the US Senate [39]. Three fifths (60%) is required to end debate for most issues, while two thirds (66%) is required for other actions. We opted for the convention used to end debate (60%). This ended up being fortuitous for timely completion of the Webinar, as there would have been six additional debates and runoff votes had we opted for a higher supermajority rate. We acknowledge that we may have received different results if we had used 66%. In the first stage of voting, a definition that received a supermajority of votes (≥60%) and also received more votes than any other definition was declared the “winner”, and the poll was advanced to the next term. When there was no clear supermajority winner, panelists discussed the definitions. Discussions were highly structured to maximize productivity during the 60-min Webinar. Panelists indicated if they wanted to make a comment by clicking a virtual hand raise button in the Webinar platform and had up to 1 min to make comments. Subsequent discussion was then limited to 5 min per strategy.

**Figure 1:**
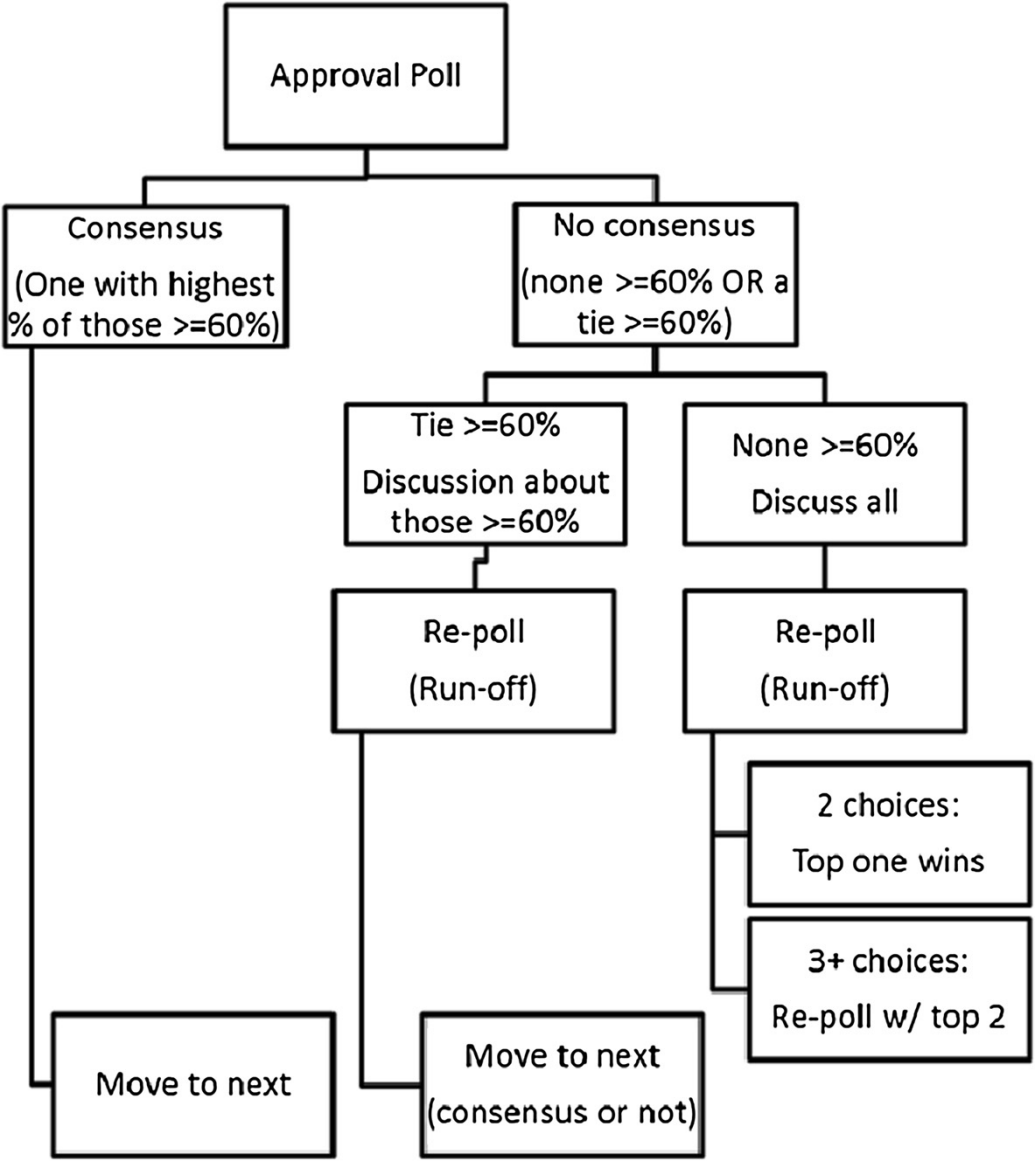
**Overview of the voting process in the final round of the modified Delphi task.** In the third and final round of the modified Delphi task, expert panelists will vote on all strategies where concerns were raised regarding the core definition in the first two online survey rounds. For each strategy, the original and proposed alternate definitions will be presented for an approval poll where participants can vote to approve all definition alternatives they find acceptable. In the first round of voting, if one definition receives a supermajority of votes (≥60%) and receives more votes than all others, that definition will be declared the winner and the poll will move to the next term. If there is no consensus, a 5-min discussion period is opened. When the discussion concludes, a runoff poll is conducted to determine the most acceptable definition alternative [13].

Following open discussion, the second stage of voting involved “runoff voting”, in which participants selected only their top choice. If only two alternatives were presented, the definition receiving the most votes was declared the winner. If three or more alternatives were presented and a majority (*i.e.*, more than 50%) was not obtained in the first runoff vote, then the top two alternatives from the first runoff round would advance to a final runoff round to determine the winner. If a tie between the original and alternative definition occurred in the runoff round, the definition already published in the literature was retained. These same voting procedures were applied to the additional strategies proposed by the expert panel in Rounds 1 and 2 of the Delphi process; however, the approval poll also included an option for the proposed strategy to be rejected if a supermajority (≥60%) of panelists deemed the strategy unworthy of inclusion. Figure 1 provides an overview of the voting process [9].

Four of the forty panelists were unable to successfully utilize the Webinar program but did participate in polling by e-mail while following the Webinar proceedings using their voting guide (Additional file 3) and participating in the discussion using the teleconference line. The multiple sources of votes (through Webinar polling and e-mails) were aggregated in real time.

The Institutional Review Board at Central Arkansas Veterans Healthcare System has approved all study procedures.

## Results

### Rounds 1 and 2

Expert panelists suggested a number of changes to Powell *et al.*’s [10] terms and definitions and proposed additional strategies. For example, suggested changes to strategy terms included changing “tailor strategies to overcome barriers and honor preferences” to simply “tailor strategies”, and “penalize” to “develop disincentives”. The alternate definition for the term “develop an implementation glossary” is illustrative of the participants’ efforts to ensure strategy definitions were clear. The original definition was “develop a glossary to promote common understanding about implementation among the different stakeholders”. A new definition was proposed, “Develop and distribute a list of terms describing the innovation, implementation, and the stakeholders in the organizational change.” Finally, five new terms and definitions were suggested in Round 1, including “promote adaptability”, “external facilitation”, “identify early adopters”, “promote network weaving”, and “provide local technical assistance”. Table 2 provides a summary of the types of changes to original strategy terms and definitions that were suggested in Rounds 1 and 2, as well as the new strategy terms that were proposed. The majority of the expert feedback received in Rounds 1 and 2 did not focus on strategy terms and core definitions, but rather involved concerns, additions, or clarifications pertaining to the ancillary material. For example, for the strategy “provide ongoing consultation”, participants noted that consultation can be conducted by individuals outside of the organization and that it can focus on system and culture change in addition to clinical concerns. Feedback on ancillary materials did not impact the core definition of the strategy and was thus integrated into the ancillary material at the discretion of the study team. A more comprehensive description of the types of feedback received in Rounds 1 and 2 can be viewed in Additional file 2.

**Table 2:** **Results from Rounds 1 and 2 of the modified Delphi process**

**Suggested changes to strategy terms and/or definitions**	**Round 1**	**Round 2**
Develop a formal implementation blueprint	2 alt	3 alt
Tailor strategies [to overcome barriers and honor preferences]	1 alt	-----
Identify and prepare champions	1 alt	-----
Involve patients/consumers and family members	1 alt	-----
Provide ongoing consultation	1 alt	-----
Shadow other clinicians	2 alt	3 alt
Change physical structure and equipment	1 alt	3 alt
Facilitate relay of clinical data to providers	1 alt	2 alt
Use advisory boards and work groups	1 alt	4 alt
Purposefully reexamine the implementation	1 alt	3 alt
Promote adaptability	New	1 alt
External facilitation	New	2 alt
Identify early adopters	New	1 alt
Promote network weaving	New	-----
Provide local technical assistance	New	-----
Assess for readiness and identify barriers *and facilitators*	Concerns	1 alt
Stage implementation scale-up	Concerns	-----
Model and simulate change	Concerns	2 alt
Mandate change	Concerns	1 alt
Develop effective educational materials	Concerns	-----
Develop *an implementation glossary* [a glossary of implementation]	Concerns	1 alt
Conduct ongoing training	Concerns	-----
Make training dynamic	Concerns	-----
Conduct educational outreach visits	Concerns	-----
Use mass media	Concerns	-----
Prepare patients/consumers to be active participants	Concerns	-----
*Develop disincentives* [penalize]	Concerns	1 alt
Create new clinical teams	Concerns	-----
Start a *dissemination* [purveyor] organization	Concerns	1 alt
Develop tools for quality monitoring	Concerns	-----
Audit and provide feedback	Concerns	-----
Use data warehousing techniques	Concerns	-----
Use an improvement/implementation advisor	Concerns	-----
Change accreditation or membership requirements	Concerns	-----
Use data experts	-----	1 alt
Use capitated payments	-----	1 alt
Organize clinician implementation team meetings	-----	1 alt
Intervene with patients/consumers to enhance uptake and adherence	-----	1 alt
Create a learning collaborative	-----	1 alt

Brackets represent elements of the strategies that have been deleted based on feedback in Rounds 1 and 2. Italicized elements were consensus modifications for the strategy.

*Alt* alternative definitions suggested.

### Round 3

The majority of the terms and definitions (69%) from the Powell *et al.* [10] compilation were considered “no contest” and were not subjected to voting in Round 3 as participants did not raise substantial concerns or suggest alternative definitions for them. Twenty-one strategies and five new strategies were subjected to voting in Round 3. The complete results from the Round 3 voting can be viewed in Additional file 5. For each vote, there was a small number of abstainers; the percentage of participants casting votes ranged from 83 to 94%. In the majority of cases, the initial vote (*i.e.*, the approval voting stage) yielded a clear winner; however, in two cases, no strategy received over 60% of the vote in the approval voting stage and in another case there was a tie between two strategies, each receiving 66% of the votes. In these situations, the participants discussed their thoughts and concerns, after which the runoff vote successfully identified a winning definition.

For the 21 alternative definitions suggested, an alternative definition was selected 81% of the time and the original definition was maintained 19% of the time. One of the advantages of approval voting was determining the acceptability of the original definitions even when alternatives were thought to be superior. In each of the 17 times in which an alternative was ultimately selected, the original definitions failed to reach the supermajority approval level of 60% (average 30%, range 3 to 51%).

Each of the five new strategies that the panel proposed was maintained in some form. Panelists had the opportunity to reject the proposed additions, but on average, across the five strategies, 84% of panelists voted to retain the new strategy (range 100 to 71%). Each of the new strategies had an initial proposed definition in Round 1. Panelists had the opportunity to suggest alternative definitions in Round 2. In two cases (“promote network weaving” and “provide local technical assistance”), no alternative definitions were proposed, and the new definition was retained with approval votes of 71 and 73%, respectively. In one case (“identify early adopters”) the alternative definition won in the approval vote. Finally, in two cases (“facilitation” and “promote adaptability”), the original new definition was selected over the alternatives in the runoff vote.

### Final compilation

The final compilation included 73 discrete strategies (Table 3). Consistent with the Powell *et al.* [10] compilation, active verbs were used to describe the implementation strategy terms. We attempted to strike a balance between economy of expression and comprehensiveness. Thus, in some cases, we used verbs like “develop” or “create” instead of “develop and implement” or “create and implement”, though the implementation or use of the strategies developed or created should be thought of as part of the same process. In many cases, this is clarified in the definition. For example, the strategy “develop a formal implementation blueprint” specifies in the definition that the blueprint should be used and updated. Each of the strategies, including those in which the verb “use” is included in the strategy term, should be thought of as discretionary for researchers and implementers. Our intent was to highlight the range of discrete strategies that could potentially be used to implement new programs and practices, not to present a checklist of strategies that must be used in all efforts. Additional file 6 contains the full compilation with ancillary material that contains additional references and details that may be useful to implementation stakeholders, such as advice about how a particular strategy might be used.

**Table 3:** **ERIC discrete implementation strategy compilation (n = 73)**

**Strategy**	**Definitions**
Access new funding	Access new or existing money to facilitate the implementation
Alter incentive/allowance structures	Work to incentivize the adoption and implementation of the clinical innovation
Alter patient/consumer fees	Create fee structures where patients/consumers pay less for preferred treatments (the clinical innovation) and more for less-preferred treatments
Assess for readiness and identify barriers and facilitators	Assess various aspects of an organization to determine its degree of readiness to implement, barriers that may impede implementation, and strengths that can be used in the implementation effort
Audit and provide feedback	Collect and summarize clinical performance data over a specified time period and give it to clinicians and administrators to monitor, evaluate, and modify provider behavior
Build a coalition	Recruit and cultivate relationships with partners in the implementation effort
Capture and share local knowledge	Capture local knowledge from implementation sites on how implementers and clinicians made something work in their setting and then share it with other sites
Centralize technical assistance	Develop and use a centralized system to deliver technical assistance focused on implementation issues
Change accreditation or membership requirements	Strive to alter accreditation standards so that they require or encourage use of the clinical innovation. Work to alter membership organization requirements so that those who want to affiliate with the organization are encouraged or required to use the clinical innovation
Change liability laws	Participate in liability reform efforts that make clinicians more willing to deliver the clinical innovation
Change physical structure and equipment	Evaluate current configurations and adapt, as needed, the physical structure and/or equipment (*e.g.*, changing the layout of a room, adding equipment) to best accommodate the targeted innovation
Change record systems	Change records systems to allow better assessment of implementation or clinical outcomes
Change service sites	Change the location of clinical service sites to increase access
Conduct cyclical small tests of change	Implement changes in a cyclical fashion using small tests of change before taking changes system-wide. Tests of change benefit from systematic measurement, and results of the tests of change are studied for insights on how to do better. This process continues serially over time, and refinement is added with each cycle
Conduct educational meetings	Hold meetings targeted toward different stakeholder groups (*e.g.*, providers, administrators, other organizational stakeholders, and community, patient/consumer, and family stakeholders) to teach them about the clinical innovation
Conduct educational outreach visits	Have a trained person meet with providers in their practice settings to educate providers about the clinical innovation with the intent of changing the provider’s practice
Conduct local consensus discussions	Include local providers and other stakeholders in discussions that address whether the chosen problem is important and whether the clinical innovation to address it is appropriate
Conduct local needs assessment	Collect and analyze data related to the need for the innovation
Conduct ongoing training	Plan for and conduct training in the clinical innovation in an ongoing way
Create a learning collaborative	Facilitate the formation of groups of providers or provider organizations and foster a collaborative learning environment to improve implementation of the clinical innovation
Create new clinical teams	Change who serves on the clinical team, adding different disciplines and different skills to make it more likely that the clinical innovation is delivered (or is more successfully delivered)
Create or change credentialing and/or licensure standards	Create an organization that certifies clinicians in the innovation or encourage an existing organization to do so. Change governmental professional certification or licensure requirements to include delivering the innovation. Work to alter continuing education requirements to shape professional practice toward the innovation
Develop a formal implementation blueprint	Develop a formal implementation blueprint that includes all goals and strategies. The blueprint should include the following: 1) aim/purpose of the implementation; 2) scope of the change (*e.g.*, what organizational units are affected); 3) timeframe and milestones; and 4) appropriate performance/progress measures. Use and update this plan to guide the implementation effort over time
Develop academic partnerships	Partner with a university or academic unit for the purposes of shared training and bringing research skills to an implementation project
Develop an implementation glossary	Develop and distribute a list of terms describing the innovation, implementation, and stakeholders in the organizational change
Develop and implement tools for quality monitoring	Develop, test, and introduce into quality-monitoring systems the right input—the appropriate language, protocols, algorithms, standards, and measures (of processes, patient/consumer outcomes, and implementation outcomes) that are often specific to the innovation being implemented
Develop and organize quality monitoring systems	Develop and organize systems and procedures that monitor clinical processes and/or outcomes for the purpose of quality assurance and improvement
Develop disincentives	Provide financial disincentives for failure to implement or use the clinical innovations
Develop educational materials	Develop and format manuals, toolkits, and other supporting materials in ways that make it easier for stakeholders to learn about the innovation and for clinicians to learn how to deliver the clinical innovation
Develop resource sharing agreements	Develop partnerships with organizations that have resources needed to implement the innovation
Distribute educational materials	Distribute educational materials (including guidelines, manuals, and toolkits) in person, by mail, and/or electronically
Facilitate relay of clinical data to providers	Provide as close to real-time data as possible about key measures of process/outcomes using integrated modes/channels of communication in a way that promotes use of the targeted innovation
Facilitation	A process of interactive problem solving and support that occurs in a context of a recognized need for improvement and a supportive interpersonal relationship
Fund and contract for the clinical innovation	Governments and other payers of services issue requests for proposals to deliver the innovation, use contracting processes to motivate providers to deliver the clinical innovation, and develop new funding formulas that make it more likely that providers will deliver the innovation
Identify and prepare champions	Identify and prepare individuals who dedicate themselves to supporting, marketing, and driving through an implementation, overcoming indifference or resistance that the intervention may provoke in an organization
Identify early adopters	Identify early adopters at the local site to learn from their experiences with the practice innovation
Increase demand	Attempt to influence the market for the clinical innovation to increase competition intensity and to increase the maturity of the market for the clinical innovation
Inform local opinion leaders	Inform providers identified by colleagues as opinion leaders or “educationally influential” about the clinical innovation in the hopes that they will influence colleagues to adopt it
Intervene with patients/consumers to enhance uptake and adherence	Develop strategies with patients to encourage and problem solve around adherence
Involve executive boards	Involve existing governing structures (*e.g.*, boards of directors, medical staff boards of governance) in the implementation effort, including the review of data on implementation processes
Involve patients/consumers and family members	Engage or include patients/consumers and families in the implementation effort
Make billing easier	Make it easier to bill for the clinical innovation
Make training dynamic	Vary the information delivery methods to cater to different learning styles and work contexts, and shape the training in the innovation to be interactive
Mandate change	Have leadership declare the priority of the innovation and their determination to have it implemented
Model and simulate change	Model or simulate the change that will be implemented prior to implementation
Obtain and use patients/consumers and family feedback	Develop strategies to increase patient/consumer and family feedback on the implementation effort
Obtain formal commitments	Obtain written commitments from key partners that state what they will do to implement the innovation
Organize clinician implementation team meetings	Develop and support teams of clinicians who are implementing the innovation and give them protected time to reflect on the implementation effort, share lessons learned, and support one another’s learning
Place innovation on fee for service lists/formularies	Work to place the clinical innovation on lists of actions for which providers can be reimbursed (*e.g.*, a drug is placed on a formulary, a procedure is now reimbursable)
Prepare patients/consumers to be active participants	Prepare patients/consumers to be active in their care, to ask questions, and specifically to inquire about care guidelines, the evidence behind clinical decisions, or about available evidence-supported treatments
Promote adaptability	Identify the ways a clinical innovation can be tailored to meet local needs and clarify which elements of the innovation must be maintained to preserve fidelity
Promote network weaving	Identify and build on existing high-quality working relationships and networks within and outside the organization, organizational units, teams, etc. to promote information sharing, collaborative problem-solving, and a shared vision/goal related to implementing the innovation
Provide clinical supervision	Provide clinicians with ongoing supervision focusing on the innovation. Provide training for clinical supervisors who will supervise clinicians who provide the innovation
Provide local technical assistance	Develop and use a system to deliver technical assistance focused on implementation issues using local personnel
Provide ongoing consultation	Provide ongoing consultation with one or more experts in the strategies used to support implementing the innovation
Purposely reexamine the implementation	Monitor progress and adjust clinical practices and implementation strategies to continuously improve the quality of care
Recruit, designate, and train for leadership	Recruit, designate, and train leaders for the change effort
Remind clinicians	Develop reminder systems designed to help clinicians to recall information and/or prompt them to use the clinical innovation
Revise professional roles	Shift and revise roles among professionals who provide care, and redesign job characteristics
Shadow other experts	Provide ways for key individuals to directly observe experienced people engage with or use the targeted practice change/innovation
Stage implementation scale up	Phase implementation efforts by starting with small pilots or demonstration projects and gradually move to a system wide rollout
Start a dissemination organization	Identify or start a separate organization that is responsible for disseminating the clinical innovation. It could be a for-profit or non-profit organization
Tailor strategies	Tailor the implementation strategies to address barriers and leverage facilitators that were identified through earlier data collection
Use advisory boards and workgroups	Create and engage a formal group of multiple kinds of stakeholders to provide input and advice on implementation efforts and to elicit recommendations for improvements
Use an implementation advisor	Seek guidance from experts in implementation
Use capitated payments	Pay providers or care systems a set amount per patient/consumer for delivering clinical care
Use data experts	Involve, hire, and/or consult experts to inform management on the use of data generated by implementation efforts
Use data warehousing techniques	Integrate clinical records across facilities and organizations to facilitate implementation across systems
Use mass media	Use media to reach large numbers of people to spread the word about the clinical innovation
Use other payment schemes	Introduce payment approaches (in a catch-all category)
Use train-the-trainer strategies	Train designated clinicians or organizations to train others in the clinical innovation
Visit other sites	Visit sites where a similar implementation effort has been considered successful
Work with educational institutions	Encourage educational institutions to train clinicians in the innovation

## Discussion

This study aimed to refine and achieve consensus on a compilation of implementation strategy terms and definitions by systematically gathering input from a wide range of stakeholders. A large, accomplished panel of implementation and clinical experts was successfully engaged in a rigorous consensus development process. Participants identified substantial concerns with 31% of the terms and/or definitions from the original Powell *et al.* [10] compilation and suggested five additional strategies. Seventy-five percent of the definitions from the original compilation were retained after voting. The expert panel achieved consensus on a final compilation of 73 implementation strategies. This study has improved the original published compilation by enhancing the clarity, relevance, and comprehensiveness of included strategies and ensuring that they resonate with a wide range of stakeholders conducting implementation research and practice.

There are several immediate uses of this compilation. First, it provides a list of discrete strategies that can serve as “building blocks” for constructing multifaceted, multilevel implementation strategies for implementation efforts or in comparative effectiveness research [4]. Second, the core definitions and ancillary materials (see Additional file 6) can be used in conjunction with available reporting guidelines [1,13,14,40,41] to improve the specification and reporting of implementation strategies in efficacy, effectiveness, and implementation research [42]. Finally, the refined compilation can be used as a tool to assess discrete strategies that have been used in published implementation research. Mazza *et al.* [18] recently demonstrated how taxonomies can be used for that purpose.

The subsequent stages of the ERIC project [9] will further enhance the utility of this compilation in a number of ways. First, expert panelists will complete concept mapping [43] and rating exercises to derive conceptually distinct categories of strategies, interrelationships between them, and a rating for each discrete strategy’s importance and feasibility. This information will help users select strategies for their planned implementation efforts by highlighting the broad categories they might consider and providing feasibility and importance ratings of both individual discrete strategies and clusters of strategies. Second, expert panels will be asked to choose the best implementation strategies to use in real-world scenarios that describe implementations of specific evidence-based practices (*e.g.*, measurement-based care for depression) in hypothetical VA mental health clinic settings that vary on certain contextual characteristics [9]. This stage of ERIC will yield recommendations about which multifaceted, multilevel strategy is best matched to specific scenarios. This information will help provide guidance for similar implementation efforts and insights into how recommendations may change based on clearly described differences in context.

As Powell *et al.* [10] cautioned, this compilation should not be thought of as a checklist. No implementation effort could feasibly utilize every one of these strategies. The ERIC compilation provides a list by which to select discrete strategies that can be used to build a tailored multicomponent strategy for implementation. Future research is needed to identify the contexts and circumstances under which each discrete strategy is effective to help guide users in their selection.

We note that while our attempt was to identify discrete strategies involving one action or process, the included strategies vary in their level of complexity. In fact, active research agendas have focused on determining the essential components of many of these “discrete” implementation strategies, such as audit and feedback [44], learning collaboratives [45], and supervision [46]. The evidence will continue to accumulate, providing more detailed specifications of components for discrete strategies to help inform future iterations of this and other compilations.

The ERIC compilation consolidated discrete implementation strategies that have been identified through other taxonomies and reviews (see Powell *et al*. [10] for a list of sources and methodological details). Thus, there are many similarities between the ERIC compilation and other taxonomies. However, the ERIC compilation addresses several limitations of previously developed taxonomies and improves upon them in three ways. First, the ERIC compilation provides clear labels and more detailed definitions for each implementation strategy. Second, it is widely applicable to implementation stakeholders in health and mental health settings (and perhaps beyond). Third, a major strength of this compilation is that it is based on consensus of a broad range of implementation experts.

There are several limitations related to the process of generating this compilation. First, had we used a different taxonomy of implementation strategies as a starting point, the modified Delphi process may have yielded different results. However, the original Powell *et al.* [10] compilation incorporated strategies from several other existing taxonomies, *e.g.*, [15-17], increasing the chances that key implementation strategies were included. The fact that the expert panelists suggested few additional strategies also increases our confidence that the compilation was relatively comprehensive. Second, the composition of our expert panel was limited to participants in North America and was mostly composed of implementation and clinical experts from the USA. This was appropriate given the ERIC project’s focus on implementing evidence-based mental health programs and practices within the VA and for pragmatic reasons (*e.g.*, scheduling the consensus meeting), but we acknowledge that broader international participation would have been ideal. This may have implications for the content of the compilation, as we discuss below. Third, it is possible that in-person meetings may have generated more nuanced discussions of strategy terms and definitions; however, the asynchronous, online process had the advantage of allowing a wide range of implementation and clinical experts to participate and also ensured anonymity of responses, which limited the possibility of participants simply yielding to the majority opinion in Rounds 1 and 2. Finally, as noted in the “Results” section, a small number of participants abstained from voting for portions of the Round 3 consensus meeting. While we can speculate as to potential reasons (*e.g.*, technical difficulties, other distractions, not finding any of the strategy terms and definitions appropriate), we cannot be certain as to why participants abstained or about whether or not this could have impacted the final results in cases in which voting results were extremely close.

There are also limitations related to the content of the refined compilation. First, the evidence base for each strategy was not considered because the purpose of this work was to identify the range of potential options available. Second, the strategies were not explicitly tied to relevant theories or conceptual models. The compilation’s utility would be enhanced by linking each strategy to the domains of prominent conceptual frameworks (*e.g.*, the Consolidated Framework for Implementation Research [47], Theoretical Domains Framework [48,49], Promoting Action on Research Implementation in Health Services (PARIHS) framework [50]). Furthermore, users might benefit from using a recently developed framework by Colquhoun and colleagues [8] to better plan use of the individual strategies by identifying: 1) active ingredients (*i.e.*, the defining characteristics of the implementation strategies); 2) causal mechanisms (*i.e.*, the processes or mediators by which strategies exert change); 3) mode of delivery or practical application (*i.e.*, the way an active ingredient is applied, such as face-to-face, Web-based, mass media, etc.); and 4) intended target (*i.e.*, the implementation strategy’s “intended effects and beneficiaries”). Lastly, while we are not aware of evidence that would suggest that the strategies in this compilation would not be applicable to many different contexts, it is possible that some of the strategies may be more applicable to US or North American settings given the focus of the ERIC project and the composition of the expert panel. Engaging a broader international panel may have revealed additional strategies that are applicable to health-care systems that are organized differently or to settings (*e.g.*, low- and middle-income countries) that are not similarly resourced. The fact that the original compilation drew from taxonomies developed in contexts other than the US, *e.g.*, [15,17] may help mitigate this potential limitation.

## Conclusions

This research advances the field by improving the conceptual clarity, relevance, and comprehensiveness of discrete implementation strategies that can be used in isolation or combination in implementation research and practice. The utility of this compilation will be extended in subsequent stages of the ERIC study. We conclude by echoing Powell *et al.*’s [10] caution that this compilation, while substantially improved, should not be viewed as the final word. We welcome further comments and critiques that will further refine this compilation and enhance its ability to inform implementation research and practice.

### Contributors

We would like to acknowledge the contributions of each member of the expert panel: Greg Aarons, University of California, San Diego; Mark Bauer, Harvard University and US Department of Veterans Affairs; Rinad Beidas, University of Pennsylvania; Sharon Benjamin, Alchemy; Ian Bennett, University of Pennsylvania; Nancy Bernardy, Dartmouth College and US Department of Veterans Affairs; Amy Bohnert, University of Michigan and US Department of Veterans Affairs; Melissa Brouwer, McMaster University; Leo Cabassa, Columbia University; Martin Charns, Boston University and US Department of Veterans Affairs; Amy Cohen, US Department of Veterans Affairs; Laurel Copeland, Scott and White Healthcare and US Department of Veterans Affairs; Torrey Creed, University of Pennsylvania; Jill Crowley, US Department of Veterans Affairs; Geoff Curran, University of Arkansas for Medical Sciences and US Department of Veterans Affairs; Laura Damschroder, University of Michigan and US Department of Veterans Affairs; Teresa Damush, Indiana University and US Department of Veterans Affairs; Afsoon Eftekhari, US Department of Veterans Affairs; Rani Elwy, Boston University and US Department of Veterans Affairs; Bradford Felker, University of Washington and US Department of Veterans Affairs; Erin Finley, University of Texas Health Science Center San Antonio and US Department of Veterans Affairs; Hildi Hagedorn, University of Minnesota and US Department of Veterans Affairs; Alison Hamilton, University of California, Los Angeles and US Department of Veterans Affairs; Susanne Hempel, RAND; Timothy Hogan, University of Massachusetts and US Department of Veterans Affairs; Bradley Karlin, Education Development Center and US Department of Veterans Affairs; Ira Katz, US Department of Veterans Affairs; Jacob Kean, Indiana University and US Department of Veterans Affairs; Shannon Kehle-Forbes, University of Minnesota and US Department of Veterans Affairs; Amy Kilbourne, University of Michigan and US Department of Veterans Affairs; Kelly Koerner, Evidence-Based Practice Institute; Sarah Krein, University of Michigan and US Department of Veterans Affairs; Julie Kreyenbuhl, University of Maryland and US Department of Veterans Affairs; Kurt Kroenke, Indiana University and US Department of Veterans Affairs; Marina Kukla, Indiana University-Purdue University Indianapolis and US Department of Veterans Affairs; Sara Landes, University of Washington and US Department of Veterans Affairs; Martin Lee, University of California, Los Angeles and Prolacta Bioscience; Cara Lewis, Indiana University-Bloomington; Julie Lowery, University of Michigan and US Department of Veterans Affairs; Brian Lund, US Department of Veterans Affairs; Aaron Lyon, University of Washington; Natalie Maples, University of Texas Health Science Center San Antonio; Stephen Marder, University of California, Los Angeles and US Department of Veterans Affairs; Monica Matthieu, Saint Louis University and US Department of Veterans Affairs; Geraldine McGlynn, US Department of Veterans Affairs; Alan McGuire, Indiana University-Purdue University Indianapolis and US Department of Veterans Affairs; Allison Metz, University of North Carolina; Amanda Midboe, US Department of Veterans Affairs; Edward Miech, Indiana University and US Department of Veterans Affairs; Brian Mittman, US Department of Veterans Affairs; Laura Murray, Johns Hopkins University; Princess Osei-Bonsu, US Department of Veterans Affairs; Richard Owen, University of Arkansas for Medical Sciences and US Department of Veterans Affairs; Louise Parker, University of Massachusetts Boston; Mona Ritchie, US Department of Veterans Affairs; Craig Rosen, Stanford University and US Department of Veterans Affairs; Anju Sahay, US Department of Veterans Affairs; Susanne Salem-Schatz, Health Care Quality Initiatives; Anne Sales, University of Michigan and US Department of Veterans Affairs; Mark Snowden, University of Washington; Leif Solberg, Health Partners; Sharon Straus, University of Toronto; Scott Stroup, Columbia University; Jane Taylor, CHAMP; Carol VanDeusen Lukas, Boston University and US Department of Veterans Affairs; Dawn Velligan, University of Texas Health Science Center San Antonio; Robyn Walser, University of California, Berkeley and US Department of Veterans Affairs; Shannon Wiltsey-Stirman, Boston University and US Department of Veterans Affairs; Gordon Wood, US Department of Veterans Affairs; Kara Zivin, University of Michigan and US Department of Veterans Affairs; and Cynthia Zubritsky, University of Pennsylvania.

## Endnote

^a^As Wensing *et al.* [51] note, the field of research focusing on “how to improve healthcare” has evolved under several different names (*e.g.,* implementation science, knowledge translation research, improvement science, research utilization, delivery science, quality improvement, etc.). While each of these traditions “bring their own nuances to the area…the reality is that there are far more commonalities in the research conducted under these different names than differences” [51]. Thus, while multiple terms may be used to describe what we define as implementation strategies (*e.g.*, knowledge translation strategies or interventions, quality improvement strategies, implementation interventions, strategies to increase research utilization, etc.), we believe that the compilation described in this paper is likely to be applicable to the research and practice occurring under these different names. Indeed, the original Powell *et al.* [10] compilation drew upon a taxonomy of “quality improvement strategies” [52] and “knowledge translation interventions”, [53] among others.

## Additional files

Additional file 1:
**Expert Recommendations for Implementing Change (ERIC) Round 1 survey for the modified Delphi.** This document contains the full survey that was administered in Round 1 of the modified Delphi process. Additional file 2:
**Round 2 of online modified Delphi.** This document contains the full survey that was administered in Round 2 of the modified Delphi process. Additional file 3:
**Expert Recommendations for Implementing Change (ERIC) voting guide.** This voting guide was mailed to participants prior to modified Delphi Round 3. Additional file 4:
**Expert Recommendations for Implementing Change (ERIC)—ballot for Round 3 of the modified Delphi process.** This ballot specifies each of the strategies that were voted on in Round 3 of the modified Delphi. Additional file 5:
**Expert Recommendations for Implementing Change (ERIC)—results from modified Delphi Round 3 voting.** This document lists the voting results from modified Delphi Round 3. Additional file 6:
**Expert Recommendations for Implementing Change (ERIC)—discrete implementation strategy compilation with ancillary material.** This file contains the final ERIC discrete strategy compilation and the associated ancillary material.

## Abbreviations

ERIC: Expert Recommendations for Implementing Change
QUERI: Quality Enhancement Research Initiative
VA: US Department of Veterans Affairs

## References

[1] Proctor EK, Powell BJ, McMillen JC (2013). Implementation strategies: recommendations for specifying and reporting. Implement Sci..

[2] Institute of Medicine (2001). Crossing the quality chasm: a new health system for the 21st century.

[3] Institute of Medicine (2006). Improving the quality of health care for mental and substance-use conditions.

[4] Institute of Medicine (2009). Initial national priorities for comparative effectiveness research.

[5] Eccles MP, Armstrong D, Baker R, Cleary K, Davies H, Davies S (2009). An implementation research agenda. Implement Sci..

[6] Michie S, Fixsen DL, Grimshaw JM, Eccles MP (2009). Specifying and reporting complex behaviour change interventions: the need for a scientific method. Implement Sci..

[7] McKibbon KA, Lokker C, Wilczynski NL, Ciliska D, Dobbins M, Davis DA (2010). A cross-sectional study of the number and frequency of terms used to refer to knowledge translation in a body of health literature in 2006: a tower of Babel?. Implement Sci..

[8] Colquhoun H, Leeman J, Michie S, Lokker C, Bragge P, Hempel S (2014). Towards a common terminology: a simplified framework of interventions to promote and integrate evidence into health practices, systems, and policies. Implement Sci..

[9] Waltz TJ, Powell BJ, Chinman MJ, Smith JL, Matthieu MM, Proctor EK (2014). Expert Recommendations for Implementing Change (ERIC): protocol for a mixed methods study. Implement Sci..

[10] Powell BJ, McMillen JC, Proctor EK, Carpenter CR, Griffey RT, Bunger AC (2012). A compilation of strategies for implementing clinical innovations in health and mental health. Med Care Res Rev..

[11] Gerring J (2001). Social science methodology: a criterial framework.

[12] Rabin BA, Brownson RC, Brownson RC, Colditz GA, Proctor EK (2012). Developing terminology for dissemination and implementation research. Dissemination and implementation research in health: translating science to practice.

[13] Albrecht L, Archibald M, Arseneau D, Scott SD (2013). Development of a checklist to assess the quality of reporting of knowledge translation interventions using the Workgroup for Intervention Development and Evaluation Research (WIDER) recommendations. Implement Sci..

[14] Davidoff F, Batalden P, Stevens D, Ogrinc G, Mooney S (2008). Publication guidelines for quality improvement in health care: evolution of the SQUIRE project. Qual Saf Health Care..

[15] Cochrane Effective Practice and Organisation of Care Group (2002). Data collection checklist.

[16] Leeman J, Baernholdt M, Sandelowski M (2007). Developing a theory-based taxonomy of methods for implementing change in practice. J Adv Nurs..

[17] Walter I, Nutley S, Davies H (2003). Developing a taxonomy of interventions used to increase the impact of research.

[18] Mazza D, Bairstow P, Buchan H, Chakraborty SP, Van Hecke O, Grech C (2013). Refining a taxonomy for guideline implementation: results of an exercise in abstract classification. Implement Sci..

[19] Bero LA, Grilli R, Grimshaw JM, Harvey E, Oxman AD, Thomson MA (1998). Getting research findings into practice: closing the gap between research and practice: an overview of systematic reviews of interventions to promote the implementation of research findings. Br Med J..

[20] Grimshaw JM, Eccles M, Thomas R, MacLennan G, Ramsay C, Fraser C (2006). Toward evidence-based quality improvement. Evidence (and its limitations) of the effectiveness of guideline dissemination and implementation strategies 1966–1998. J Gen Intern Med.

[21] Grol R, Grimshaw JM (2003). From best evidence to best practice: effective implementation of change in patients’ care. Lancet..

[22] Shojania KG, Ranji SR, McDonald KM, Grimshaw JM, Sundaram V, Rushakoff RJ (2006). Effects of quality improvement strategies for type 2 diabetes on glycemic control: a meta-regression analysis. JAMA..

[23] Cabana MD, Rushton JL, Rush J (2002). Implementing practice guidelines for depression: applying a new framework to an old problem. Gen Hosp Psychiatry..

[24] Gilbody S, Whitty P, Grimshaw JM, Thomas R (2003). Educational and organizational interventions to improve the management of depression in primary care: a systematic review. JAMA..

[25] Stone EG, Morton SC, Hulscher ME, Maglione MA, Roth EA, Grimshaw JM (2002). Interventions that increase use of adult immunization and cancer screening services: a meta-analysis. Ann Intern Med..

[26] Hysong SJ, Best RG, Pugh JA (2007). Clinical practice guideline implementation strategy patterns in veterans affairs primary care clinics. Health Serv Res..

[27] Magnabosco JL (2006). Innovations in mental health services implementation: a report on state-level data from the U.S. evidence-based practices project. Implement Sci.

[28] Katon WJ, Zatzick D, Bond G, Williams J (2006). Dissemination of evidence-based mental health interventions: importance to the trauma field. J Trauma Stress..

[29] McHugh RK, Barlow DH (2010). The dissemination and implementation of evidence-based psychological treatments. Am Psychol..

[30] Ryan R, Lowe D, Santesso N, Hill S (2010). Development of a taxonomy of interventions directed at consumers to promote evidence-based prescribing and medicines use: a tool for evidence-based decision-making.

[31] Raghavan R, Bright CL, Shadoin AL (2008). Toward a policy ecology of implementation of evidence-based practices in public mental health settings. Implement Sci..

[32] Mathew D, McKibbon KA, Lokker C, Colquhoun H (2014). Engaging with a wiki related to knowledge translation: a survey of WhatisKT Wiki user. J Med Internet Res.

[33] Palinkas LA, Horwitz SM, Green CA, Wisdom JP, Duan N, Hoagwood K (2013). Purposeful sampling for qualitative data collection and analysis in mixed method implementation research. Adm Policy Ment Health.

[34] Stetler CB, Mittman BS, Francis J (2008). Overview of the VA Quality Enhancement Research Initiative (QUERI) and QUERI theme articles: QUERI series. Implement Sci..

[35] Proctor EK, Landsverk J, Baumann AA, Mittman BS, Aarons GA, Brownson RC (2013). The implementation research institute: training mental health implementation researchers in the United States. Implement Sci..

[36] Hasson F, Keeney S (2011). Enhancing rigor in the Delphi technique research. Technol Forecast Soc Change..

[37] Fishburn PC, Brams SJ (1981). Expected utility and approval voting. Syst Res Behav Sci..

[38] Brams SJ, Fishburn PC (1978). Approval voting. Am Polit Sci Rev..

[39] Oleszek WJ (2008). Super-majority votes in the Senate.

[40] WIDER recommendations to improve reporting of the content of behaviour change interventions [http://www.implementationscience.com/content/supplementary/1748-5908-7-70-s4.pdf]

[41] Davidoff F, Batalden P (2005). Toward stronger evidence on quality improvement. Draft publication guidelines: the beginning of a consensus project. Qual Saf Health Care.

[42] Proctor EK, Rosen A (2008). From knowledge production to implementation: research challenges and imperatives. Res Soc Work Pract..

[43] Kane M, Trochim WMK (2007). Concept mapping for planning and evaluation.

[44] Ivers NM, Sales A, Colquhoun H, Michie S, Foy R, Francis JJ (2014). No more “business as usual” with audit and feedback interventions: towards an agenda for a reinvigorated intervention. Implement Sci..

[45] Nadeem E, Olin S, Hoagwood KE, Horwitz SM (2013). Understanding the components of quality improvement collaboratives: a systematic literature review. Milbank Q..

[46] Dorsey S, Pullman MD, Deblinger E, Berliner L, Kerns SE, Thompson K (2013). Improving practice in community-based settings: a randomized trial of supervision—study protocol. Implement Sci..

[47] Damschroder LJ, Aron DC, Keith RE, Kirsh SR, Alexander JA, Lowery JC (2009). Fostering implementation of health services research findings into practice: a consolidated framework for advancing implementation science. Implement Sci..

[48] Michie S, Johnston M, Abraham C, Lawton R, Parker D, Walker A (2005). Making psychological theory useful for implementing evidence based practice: a consensus approach. Qual Saf Health Care..

[49] Cane J, O’Connor D, Michie S (2012). Validation of the theoretical domains framework for use in behaviour change and implementation research. Implement Sci..

[50] Rycroft-Malone J (2004). The PARiHS framework: a framework for guiding the implementation of evidence-based practice. J Nurs Care Qual..

[51] Wensing M, Grimshaw JM, Eccles MP (2012). Does the world need a scientific society for research on how to improve healthcare?. Implement Sci..

[52] Shojania KG, McDonald KM, Wachter RM, Owens DK (2004). Closing the quality gap: a critical analysis of quality improvement strategies, volume 1—series overview and methodology. Technical review 9.

[53] Wensing M, Bosch M, Grol R, Straus S, Tetroe J, Graham ID (2009). Selecting, tailoring, and implementing knowledge translation interventions. Knowledge translation in health care: moving from evidence to practice.

