# Supplementary material for: A refined compilation of implementation strategies: results from the Expert Recommendations for Implementing Change (ERIC) project
Source: Implement Sci. 2015 Feb 12;10:21. doi: 10.1186/s13012-015-0209-1 (PMC4328074; doi:10.1186/s13012-015-0209-1)
Supplement: Additional file 1: — Expert Recommendations for Implementing Change (ERIC) Round 1 survey for the modified Delphi. This document contains the full survey that was administered in Round 1 of the modified Delphi process. [file 13012_2015_209_MOESM1_ESM.docx]

**Additional File 1:**

Expert Recommendations for Implementing Change (ERIC)

Round 1 Survey for the Modified-Delphi

SECTION 1:

The table below lists a number of discrete implementation strategies along with their definitions. For the purposes of this exercise, discrete implementation strategies are defined as single actions or processes that may be used to support implementation of a given evidence­based practice or clinical innovation. The discrete implementation strategies listed below were taken from: Powell, B.J., McMillen, J.C., Proctor, E.K., Carpenter, R.C., Griffey, R.T., Bunger, A.C., Glass, J.E., & York, J.L. (2012). A compilation of strategies for implementing clinical innovations in health and mental health. Medical Care Research and Review, 69, 123­157.

Before reviewing these terms, take a moment and think of all the implementation projects with which you are most familiar. Taking all of these experiences into consideration, please review the list of discrete implementation strategies below.

If a listed strategy is very similar to other strategies (by a different name) with which you are familiar, please enter the names of the similar strategy(ies) in the “Synonyms” text box. If you have any additional thoughts or concerns regarding the definition provided for a given implementation strategy (e.g., specificity, breadth, or deviation from a familiar source), please type those comments into the “Comments” text box.

**Conduct local needs assessment**

Collect and analyze data related to the need for the innovation; this assessment could be focused on the description of usual care and its distance from evidence based care, outcomes of usual care, opinions from stakeholders on the needs for an innovation, or on special considerations for delivering the innovation in the local context.

Synonyms:

Comments:

**Assess for readiness & identify barriers**

Assess various aspects of an organization to determine its degree of readiness to implement, barriers that may impede implementation, and strengths that can be used in the implementation effort. The assessment may focus on agency finances, other services provided, community support, clinician attitudes and beliefs, organizational climate and culture, structure, and decision-making styles. There are also specific measures created to assess readiness to change that could be helpful (e.g., Lehman, Greener, & Simpson, 2002; Weiner, Amick, & Lee, 2008). The readiness assessment can be used to vet or eliminate implementation sites.

Synonyms:

Comments:

**Visit other sites**

Visit sites where a similar implementation effort has been considered successful.

Synonyms:

Comments:

**Develop a formal implementation blueprint**

Develop a formal implementation blueprint that integrates multiple strategies from multiple levels or domains (e.g., staffing, funding, monitoring) using multiple theories or the use of an explicit theoretical framework. Use and update this plan to guide the implementation effort over time.

Synonyms:

Comments:

**Tailor strategies to overcome barriers and honor preferences**

Tailor the implementation effort to address barriers and to honor stakeholder preferences that were identified through earlier data collection.

Synonyms:

Comments:

**Stage implementation scale up**

Phase implementation efforts by starting with small pilots or demonstration projects and gradually moving to system wide rollout.

Synonyms:

Comments:

**Model and simulate change**

Model or simulate the change that will be implemented prior to implementation. These efforts could involve computer simulations, walkthrough simulation exercises, or modeling the overall impact of clinicians’ intentions to change their clinical behaviors.

Synonyms:

Comments:

**Conduct local consensus discussions**

Include providers and other stakeholders in discussions that address whether the chosen problem is important and whether the clinical innovation to address it is appropriate.

Synonyms:

Comments:

**Involve executive boards**

Involve existing governing structures (e.g., boards of directors, medical staff boards of governance) in the implementation effort, including the review of data on implementation processes.

Synonyms:

Comments:

**Identify and prepare champions**

Cultivate relationships with people who will champion the clinical innovation and spread the word of the need for it. This strategy includes preparing individuals for their role as champions. Champions can be internal or external to the organization.

Synonyms:

Comments:

**Involve patients/consumers and family members**

Engage or include patients/consumers and families in all phases of the implementation effort, including training in the clinical innovation, and advocacy related to the innovation effort.

Synonyms:

Comments:

**Recruit, designate, and train for leadership**

Recruit, designate, and train leaders for the change effort. Change efforts require certain types of leaders, and organizations may need to recruit accordingly, rather than assuming that their current personnel can implement the change. Designated change leaders can include an executive sponsor and a day-to-day manager of the effort.

Synonyms:

Comments:

**Mandate change**

Declare that the innovation will be implemented.

Synonyms:

Comments:

**Build a coalition**

Recruit and cultivate relationships with partners in the implementation effort. Partnerships can develop around cost-sharing, shared resources, shared training, and the division of responsibilities among partners. This work may proceed naturally from local consensus discussions.

Synonyms:

Comments:

**Develop resource-sharing agreements**

Develop partnerships with organizations that have resources needed to implement the innovation. As an example, a group of providers could strike a relationship with a microbiology lab to conduct specialized lab work needed to implement an innovation efficiently.

Synonyms:

Comments:

**Obtain formal commitments**

Obtain written commitments from key partners that state what they will do to implement the innovation.

Synonyms:

Comments:

**Develop academic partnerships**

Partner with a university or academic unit for the purposes of shared training and bringing research skills to an implementation project.

Synonyms:

Comments:

**Develop effective educational materials**

Develop and format guidelines, manuals, toolkits and other supporting materials in ways that make it easier for stakeholders to learn about the innovation and for clinicians to learn how to deliver the clinical innovation. Create eye-catching, easy-to-use documents. Distill complex information into easier-to-learn components. Consider teaching skills modularly. Use different forms of media. Target messages for different audiences.

Synonyms:

Comments:

**Develop a glossary of implementation**

Develop a glossary to promote common understanding about implementation among the different stakeholders.

Synonyms:

Comments:

**Distribute educational materials**

Distribute educational materials (including guidelines, manuals and toolkits) in person, by mail, and/or electronically.

Synonyms:

Comments:

**Conduct educational meetings**

Hold meetings targeted toward providers, administrators, other organizational stakeholders, and community, patient/consumer, and family stakeholders to teach them about the clinical innovation.

Synonyms:

Comments:

**Conduct ongoing training**

Plan for and conduct training in the clinical innovation in an ongoing way. This can include follow-up training, advanced training, booster training, purposefully spaced training, training to competence, integration of off the- job and on-the-job training, the introduction of concepts in a specific sequence to ensure mastery, and trainings based on the level of clinician knowledge. Trainings can be in-person, on the web, or technology-assisted.

Synonyms:

Comments:

**Make training dynamic**

Vary the information delivery methods to cater to different learning styles and work contexts, and shape the training in the innovation to be interactive. This includes efforts to divide material into small time intervals and the use of small group breakouts, audience response systems, and other measures.

Synonyms:

Comments:

**Conduct educational outreach visits**

Use a trained person who meets with providers in their practice settings to educate providers about the clinical innovation with the intent of changing the provider’s practice. The term academic detailing is often used synonymously.

Synonyms:

Comments:

**Use train-the-trainer strategies**

Train designated clinicians or organizations to train others in the clinical innovation. Determine whether clinicians trained as trainers are eligible to train others as train the trainers.

Synonyms:

Comments:

**Provide ongoing consultation**

Provide clinicians with continued consultation with an expert in the clinical innovation. This could include in-person or distance consultation and feedback on taped clinical encounters. This consultation is tailored to the clinician’s actual practice, to differentiate it from ongoing training. This feedback may be from a consultant external to the organization, which distinguishes it from clinical supervision.

Synonyms:

Comments:

**Inform local opinion leaders**

Inform providers identified by colleagues as opinion leaders or “educationally influential” about the clinical innovation in the hopes that they will influence colleagues to adopt it.

Synonyms:

Comments:

**Create a learning collaborative**

Develop and use groups of providers or provider organizations that will implement the clinical innovation and develop ways to learn from one another to foster better implementation. This is called several things in the literature including peer consultation networks, online communities of practice, quality circles, and learning collaboratives.

Synonyms:

Comments:

**Shadow other clinicians**

Have clinicians shadow other clinicians who are experts or knowledgeable in the clinical innovation and have implemented it.

Synonyms:

Comments:

**Use mass media**

Use media to reach large numbers of people to spread the word about the clinical innovation.

Synonyms:

Comments:

**Prepare patients/consumers to be active participants**

Prepare patients/consumers to be active in their care, to ask questions, and specifically to inquire about care guidelines, the evidence behind clinical decisions, or about available evidence-supported treatments.

Synonyms:

Comments:

**Increase demand**

Attempt to influence the market for the clinical innovation to increase competition intensity and to increase the maturity of the market for the clinical innovation.

Synonyms:

Comments:

**Work with educational institutions**

Encourage educational institutions to train clinicians in the innovation.

Synonyms:

Comments:

**Alter incentive/ allowance structures**

Work to incent the adoption and implementation of the clinical innovation. The incentive could be in the form of an increased rate of pay to cover the incremental costs associated with implementing the clinical innovation. The incentive could be through loan reduction/forgiveness to clinicians as an incentive to learn an innovation. This category of financial strategies also includes the elimination of any perverse incentives (incentives that become a barrier to receiving appropriate care). An incentive suggests the payment is tied to performing the clinical action. An allowance suggests that the clinician is not required to perform the clinical action.

Synonyms:

Comments:

**Use capitated payments**

Pay providers a set amount per patient/consumer for delivering clinical care. This is an implementation strategy to the degree that it frees the clinician to provide services that they may have been disincentivized to provide under a fee-for-service structure. This may be helpful to motivate clinicians to use certain clinical innovations.

Synonyms:

Comments:

**Penalize**

Penalize providers financially for failure to implement or use the clinical innovation.

Synonyms:

Comments:

**Use other payment schemes**

Introduce payment approaches (in a catch-all category) such as prepayment and prospective payment for service, provider salaried service, the alignment of payment rates with the attainment of patient/consumer outcomes, and the removal or alteration of billing limits (such as numbers of encounters that are reimbursable). These are implementation strategies to the degree that they free the clinician to provide the clinical innovation. Others motivate the clinician to provide better service.

Synonyms:

Comments:

**Reduce or increase patient/consumer fees**

Create fee structures where patients/consumers pay less for preferred treatments (the clinical innovation) and more for less-preferred treatments.

Synonyms:

Comments:

**Place on fee for service lists/formularies**

Work to place the clinical innovation on lists of actions for which providers can be reimbursed (e.g., a drug is placed on a formulary, a procedure is now reimbursable).

Synonyms:

Comments:

**Fund and contract for the clinical innovation**

(Governments and other payers of services) issue requests for proposals to deliver the innovation, use contracting processes to motivate providers to deliver the clinical innovation, and develop new funding formulas that make it more likely that providers will deliver the innovation.

Synonyms:

Comments:

**Access new funding**

Access new or existing money to facilitate the implementation. This could involve new uses of existing money, accessing block grants, shifting funding from one program to another, cost-sharing, passing new taxes, raising private funds, or applying for grants.

Synonyms:

Comments:

**Make billing easier**

Make it easier to bill for the clinical innovation. This might involve requiring less documentation, “block” funding for delivering the innovation, and creating new billing codes for the innovation.

Synonyms:

Comments:

**Revise professional roles**

Shift and revise roles among professionals who provide care and redesign job characteristics. This includes the expansion of roles to cover provision of the clinical innovation and the elimination of service barriers to care, including personnel policies.

Synonyms:

Comments:

**Create new clinical teams**

Change who serves on the clinical team, adding different disciplines and different skills to make it more likely that the clinical innovation is delivered or more successful.

Synonyms:

Comments:

**Change service sites**

Change the location of clinical service sites to increase access; this includes colocating different services to better implement complex clinical innovations that require multiple disciplines or services.

Synonyms:

Comments:

**Change physical structure and equipment**

Change the physical structure and equipment (changing the layout of a room, adding equipment).

Synonyms:

Comments:

**Facilitate relay of clinical data to providers**

Collect new clinical information from the patient/consumer and relay it to the provider outside the traditional clinical encounter to prompt the provider to use the clinical innovation. Examples might include depression scores from an instrument administered in the waiting room or telephone transmission of blood pressure measurements.

Synonyms:

Comments:

**Change records systems**

Change records systems to allow better assessment of implementation or of outcomes of the implementation.

Synonyms:

Comments:

**Start a purveyor organization**

Start a separate organization that is responsible for disseminating the clinical innovation. It could be a for-profit or nonprofit organization. It could be “licensed” by a university if the innovation was born within an academic setting.

Synonyms:

Comments:

**Develop and organize quality monitoring systems**

Develop and organize systems and procedures that monitor clinical processes and/or outcomes for the purpose of quality assurance and improvement. This includes developing systems for monitoring through peer reviews, collecting data from patients/consumers, clinicians, and supervisors, and using administrative and electronic record data. This category of strategies also includes the design of disease-specific clinical registries, where clinical information and tools (graphical representations, real-time report cards, comparisons with benchmarks, etc.) are available to care team members. These systems may inform audit and feedback strategies.

Synonyms:

Comments:

**Develop tools for quality monitoring**

Develop, test, and introduce into quality-monitoring systems the right input—the appropriate language, protocols, algorithms, standards, and measures (of processes, patient/consumer outcomes, and implementation outcomes) that are often specific to the innovation being implemented.

Synonyms:

Comments:

**Audit and provide feedback**

Collect and summarize clinical performance data over a specified time period and give it to clinicians and administrators in the hopes of changing provider behavior. The summary may include recommendations. The information may have been obtained from a variety of sources, including medical records, computerized databases, observation, or feedback from patients. A performance evaluation could also be considered as audit and feedback if it included specific information on clinical performance.

Synonyms:

Comments:

**Remind clinicians**

Develop reminder systems designed to prompt clinicians to recall information or use the clinical innovation. The reminder could be patient or encounter specific, provided verbally, on paper, or on a computer screen. Computer-aided decision support and drug dosages are included in this strategy.

Synonyms:

Comments:

**Use advisory boards & work groups**

Involve multiple kinds of stakeholders in a group to oversee implementation efforts and make recommendations.

Synonyms:

Comments:

**Obtain and use patient/consumer and family feedback**

Use mechanisms to increase patient/consumer and family feedback on the implementation effort. This could include complaint forms or methods to funnel feedback to advisory boards.

Synonyms:

Comments:

**Centralize technical assistance**

Develop and use a system to deliver technical assistance focused on implementation issues. This could be the designation of a lead technical assistance organization (could also be responsible for training). The lead technical assistance entity can develop other mechanisms (e.g., call-in lines or websites) in order to share information on how to best implement the clinical innovation.

Synonyms:

Comments:

**Provide clinical supervision**

Provide clinicians with ongoing supervision. Provide training for clinical supervisors who will supervise clinicians who provide the innovation.

Synonyms:

Comments:

**Intervene with patients/consumers to enhance uptake and adherence**

Intervene with patients/consumers to increase uptake of and adherence to clinical treatments. This includes consumer/patient reminders and financial incentives to attend appointments.

Synonyms:

Comments:

**Purposefully reexamine the implementation**

Obtain commitment from stakeholders to use monitoring to adjust practice and strategies to continuously improve the implementation effort and delivery of the clinical innovation.

Synonyms:

Comments:

**Conduct cyclical small tests of change**

Implement changes in a cyclical fashion using small tests of change before taking changes system wide. Results of the tests of change are studied for insights on how to do better. This process continues serially over time and refinement is added with each cycle. Two common small tests of change cycling strategies are “Plan-Do-Study-Act” from Deming’s quality management work and six sigma’s Define- Measure-Analyze-Improve-Control sequence.

Synonyms:

Comments:

**Use data warehousing techniques**

Integrate clinical records across facilities and organizations to facilitate implementation across systems.

Synonyms:

Comments:

**Use an improvement/implementation advisor**

Seek guidance from experts in implementation. This could include consultation with outside experts such as university-affiliated faculty members, or hiring quality improvement experts or implementation professionals.

Synonyms:

Comments:

**Use data experts**

Involve, hire, and/or consult experts in data management to shape use of the considerable data that implementation efforts can generate.

Synonyms:

Comments:

**Capture and share local knowledge**

Capture local knowledge from implementation sites on how implementers and clinicians made something work in their setting and then share it with other sites (see, centralized technical assistance and learning collaboratives).

Synonyms:

Comments:

**Organize clinician implementation team meetings**

Develop and support teams of clinicians who are implementing the innovation and give them protected time to reflect on the implementation effort, share lessons learned, and support one another’s learning.

Synonyms:

Comments:

**Change accreditation or membership requirements**

Strive to alter accreditation standards so that they require or encourage use of the clinical innovation. Work to alter membership organization requirements so that those who want to affiliate with the organization are encouraged or required to use the clinical innovation.

Synonyms:

Comments:

**Change liability laws**

Participate in liability reform efforts that make clinicians more willing to deliver the clinical innovation.

Synonyms:

Comments:

**Create or change credentialing and/or licensure standards**

Create an organization that certifies clinicians in the innovation or encourages an existing organization to do so. Change governmental professional certification or licensure requirements to include delivering the innovation. Work to alter continuing education requirements to shape professional practice toward the innovation.

Synonyms:

Comments:

SECTION 2:

Again considering all of your experiences with implementation initiatives, and considering the list of discrete implementation strategies above from Powell, et al. (2012), can you think of any additional strategies that were not included in the list? If so, please provide the name of the strategy below and provide a definition (with reference citation) for the strategy. If you feel the list of terms in Section 1 was adequately comprehensive, you can leave this section blank.

**Additional Strategy 1:**

**Additional Strategy 2:**

**Additional Strategy 3:**

**Additional Strategy 4:**

**Additional Strategy 5:**
