# Supplementary material for: A refined compilation of implementation strategies: results from the Expert Recommendations for Implementing Change (ERIC) project
Source: Implement Sci. 2015 Feb 12;10:21. doi: 10.1186/s13012-015-0209-1 (PMC4328074; doi:10.1186/s13012-015-0209-1)
Supplement: Additional file 2: — Round 2 of online modified Delphi. This document contains the full survey that was administered in Round 2 of the modified Delphi process. [file 13012_2015_209_MOESM2_ESM.pdf]

Additional File 2

Round 2 of online modified-Delphi

Welcome to ERIC modified Delphi Round 2.

The ERIC investigative team would like to thank everyone who participated in Round 1. An incredible 77% of invitees responded to the invitation and made contributions.

In Round 1, the text describing the core definition and ancillary material for each strategy were presented as continuous text. In Round 2, the ancillary material is presented separate from the core definition. This is intended to ease the burden of the review and commentary process. The core definition will be fixed by the end of the modified Delphi activities. Ancillary material is intended to be more fluid and accommodate additional guidance and examples regarding enacting the strategy. We expect to aggregate additional ancillary material across the ERIC activities and after the formal activities are completed.

> Note that ERIC Part 2 activities (i.e., Menu-Based Choice and consensus webinar) will provide further opportunities for respondents to contribute ancillary material specific to the three practice changes included in those activities. Thus, one strategy for expediting your participation in Round 2 is to reserve ancillary material additions for the Part 2 activities. However, ancillary material comments are still welcome in Round 2.

In Round 2, the strategies are organized by section based on the types of feedback received in Round 1. An overview of the sections is provided below.

- <u>Section 2.</u> **Terms targeted for voting**. This section contains strategies where respondents provided alternate definitions or other content that will likely be involved in the voting conducted in the consensus webinar. Many of these terms also received ancillary material contributions.
- <u>Section 3.</u> **Terms where the core definition raised concerns.** This section contains strategies where respondents noted possible difficulties with the core definition but no alternate definitions were proposed. Many of these terms also received ancillary material contributions.
- <u>Section 4</u>. **Terms with no comments about the core definition or ancillary materials.** This section contains strategies where none of the comments suggested changes to the core definition or ancillary material.
- <u>Section 5.</u> **Terms with few comments on ancillary materials.** This section contains strategies where two or fewer respondents suggested ancillary material changes. No changes to the core definition were suggested.
- <u>Section 6.</u> **Terms with many comments on ancillary materials.** This section contains strategies where three or more respondents suggested ancillary material changes. No changes to the core definition were suggested.
- <u>Section 7.</u> The study information sheet for the ERIC project is provided. This is the same information sheet you received when you were invited to participate in the study. The “Done” button is at the bottom of this section.

Notes on how Round 1 feedback regarding the strategy descriptions was integrated:

Comments were triaged as concerning either a) the core definition b) ancillary material, or c) other.

- **Core definition comments**:
    - Comments identifying substantive changes to the definition resulted in proposed alternate definitions, labeled as ALT 1, ALT 2, etc. Terms with these types of suggestions are listed in Section 2.
    - Comments noting concerns with the definition (e.g., clarity, wordsmithing) are listed in Section 3.
    - Note, suggestions regarding merging terms with similar content were noted but no actual merging will occur as part of the modified Delphi process. The Concept Mapping activity will provide an empirical basis for collapsing strategies later in this project.

- **Ancillary material comments** were integrated into the ancillary material narrative.

- **Other comments** contributed to the total comment count provided in the summary data for each strategy. The content of these comments are not included in the summary.
    - Some of these comments were editorial.
    - Incorporation of comments regarding the evidence base or feasibility of the strategies is beyond the scope of this project.
    - Comments that would require the investigators to research the specific details of implementation frameworks were not followed up upon due to time restraints. ERIC investigative team members are interested in relating the strategies included in this project to existing implementation frameworks and these relationships will be explored at another date. In many cases, the source paper for the strategies presented in ERIC (Powell et al., 2012) had referenced the frameworks/approaches suggested (e.g., EPOC). If you feel that an important discrete implementation strategy had been obscured through the compilation process in Powell et al. (2012) please clearly specify the strategy and its definition in your comments in Round 2. The last comment box in Section 2 is for these proposals.

Synonym data has been retained for future data analysis. Only in instances where the label for a strategy directly conflicted with the existing literature were alternate labels proposed (in Section 2). Terms where the relationship between the label and the definition were of concern are included in Section 3.

2. Terms targeted for voting.

Comments to these terms involved suggestions for alternative definitions and there was one instance of a strategy label being too specific and a more general label is desired.

This is your opportunity to make further comments or alternate definition suggestions regarding these strategies prior to the consensus webinar where voting will occur.

If you feel an alternative definition for the strategy is warranted, propose one in the respective comment box or endorse one of the alternate (ALT) definitions that are listed below the strategy's Round 1 summary by using the comment box.

If you feel the original definition for the strategy is adequate, you can leave the respective comment box empty.

Ancillary material contributions are welcome but not necessary.

****Reminder. Many Round 1 comments noted the similarity or overlap of many of the strategies. Concept Mapping data from a later phase in this study will be used to provide a quantitative basis for considering the merging of terms.

Develop a formal implementation blueprint

Develop a formal implementation blueprint that integrates multiple strategies from multiple levels or domains (e.g., staffing, funding, monitoring) using multiple theories or the use of an explicit theoretical framework. Use and update this plan to guide the implementation effort over time.

Ancillary Materials:
The blueprint/manual may be informed by one or more theories or conceptual frameworks and data from pre-implementation assessments. This document can also provide a useful historical record of the implementation process when changes are tracked over time. It is often useful to ensure you receive feedback from prospective frontline users of the blueprint prior to implementation.
Consider coordinating this strategy with developing a fidelity monitoring tool.
Examples of projects using a blueprint include:
HI-TIDES (Pyne et al., 2011)
Depression in substance abusing population (Curran et al., 2008)
Fortney NIMH RISP (reference)

"Summary:
14 of 57 respondents made comments.
5 of 57 comments concerned the core definition.
3 of 57 respondents suggested the core definition was too complicated and unnecessarily committed to the use of multiple strategies from multiple levels. ALT 1 is intended to addresses these concerns.
1 of 57 respondents requested a more prescriptive core definition specifying that the blueprint should be based on relevant theory and the results of pre-implementation assessments. ALT 2 is intended to address these concerns.
1 of 57 suggested referring to ""plan"" instead of ""blueprint""
6 of 57 made comments focused on ancillary material
1 of 57 inquired whether blueprints were for the overall implementation or if they could also be site specific"

ALT 1: Develop a formal implementation blueprint. Use and update this plan to guide the implementation effort over time.

ALT 2: Develop a formal implementation blueprint that integrates multiple strategies from multiple levels or domains (e.g., staffing, funding, monitoring) using relevant theory and the results of pre-implementation barrier/facilitator assessments. Use and update this plan to guide the implementation effort over time.

Comments

Tailor strategies to overcome barriers and honor preferences

Tailor the implementation effort to address barriers and to honor stakeholder preferences that were identified through earlier data collection.

Ancillary Materials:
The tailoring process tends to be idiosyncratic. It is important to identify the core components of the innovation and implementation strategies that are required to maintain fidelity and effectiveness and which components are amenable to modification/adaptation.

Summary:
16 of 57 respondents made comments.
6 of 57 comments concerned the core definition.
1 of 57 recommended speaking of implementation "strategies" rather than implementation "efforts" in the core definition
1 of 57 recommended using "accommodate" rather than "honor" as honoring may interfere with implementing essential aspects of the change
1 of 57 suggested dividing the definition into two concepts: tailoring to address a) barriers and b) stakeholder preferences
2 of 57 respondents indicated that this is integral to the blueprint activity and may not be needed as a separate strategy
1 of 57 indicated that this is more of a general principle than a strategy
ALT 1 represents an alternative definition developed from the core definition comments above
6 of 57 made comments focused on ancillary material

ALT 1: Label change:"Tailor strategies"
Tailor the implementation strategies to address barriers and leverage facilitators that were identified through earlier data collection.
Ancillary materials would be amended to note the accommodation of stakeholder preferences (within the barrier/facilitator assessment framework) while maintaining focus on delivering the critical components of the innovation to be implemented.

Comments

Identify and prepare
champions

Cultivate relationships with people who will champion the clinical innovation and spread the word of the need for it.

Ancillary Materials:
This strategy includes preparing individuals for their role as champions. Champions are primarily internal to the organization. Additional issues raised include the need for guidance regarding:
a) methods and considerations related to the selection/identification of champions
b) training and or providing champions support materials
c) addressing incentives/disincentives to the champion role
d) whether there are needs for champions at different levels of an organization (e.g., clinic, region, national)

Summary:
14 of 57 of respondents made comments
4 of 57 commented on the core definition
3 of 57 suggested the core definition provide more guidance regarding how to identify and prepare champions
1 of 57 suggested an alternate definition from the published literature. See ALT 1.
6 of 57 indicated needs for substantial ancillary materials

ALT 1:
Individuals who dedicate themselves to supporting, marketing, and driving through an implementation, overcoming indifference or resistance that the intervention may provoke in an organization. (Damschroeder et al., 2009)

Comments

Involve patients/
consumers and
family members

Engage or include patients/consumers and families in all phases of the implementation effort, including training in the clinical innovation, and advocacy related to the innovation effort.

Ancillary Material:
Feedback from these stakeholders can be obtained at any stage of the implementation process depending on the needs and goals of project.

Summary:
8 of 57 made comments
4 of 57 commented on the core definition
3 of 57 indicated that consumers are just another stakeholder group and a single strategy inclusive of all stakeholder groups would be more desirable. Note: the Concept Mapping task will be the tool used to identify strategies that can be collapsed together.
1 of 57 indicated the core definition was overly broad and that including consumers in all phases is not always practical or advisable. ALT 1 definition is intended reflect this suggestion.
1 of 57 respondents made comments regarding ancillary content

ALT 1:
Engage or include patients/consumers and families in the implementation effort

Comments

Provide ongoing
consultation

Provide clinicians with continued consultation with an expert in the clinical innovation.

Ancillary Material:
This could include in-person or distance consultation and feedback on taped clinical encounters. This consultation is tailored to the clinician's actual practice, to differentiate it from ongoing training. This feedback may be from a consultant external to the organization, which distinguishes it from clinical supervision.
Some practice changes can involve a recertification process involving consultation to ensure adequate fidelity.

Summary:
6 of 57 respondents made comments
4 of 57 noted concerns with the core definition.
2 of 57 noted overlap between this concept an "conduct ongoing training" or "conduct educational outreach visits"
1 of 57 noted that supervision can be conducted by persons external to the organization
1 of 57 noted that consultation can also focus on system and culture change: ALT 1
1 of 57 provided ancillary material

ALT 1:
Provide ongoing consultation with an expert in the strategies used to support implementing the innovation. Consultation activities may focus on system change, culture change, or clinical skills.

Comments

Create a learning
collaborative

Develop and use groups of providers or provider organizations that will implement the clinical innovation and develop ways to learn from one another to foster better implementation.

Ancillary Material:
There are several approaches to this in the literature including peer consultation networks, online communities of practice, quality circles, and learning collaboratives. Positive deviance approaches use Discovery and Action Dialogue among peers to promote collaborative learning. [citations for the specific approaches can be made at a later date] Groups may meet in person or interact using a wide variety of media.

Summary:
7 of 57 respondents made comments
1 of 57 suggested changing the label for this strategy noting that a "learning collaborative" is a very specific methodology pioneered by the Institute for Health Care Improvement. This term and its definition intends to describe a larger construct.
2 of 57 respondents made ancillary material contributions

Alternate Labels:
Create a learning community
Create a learning network
Create a community of practice

Comments

Shadow other
clinicians

Have clinicians shadow other clinicians who are expert or knowledgeable in the clinical innovation and have implemented it.

Summary:
5 of 57 respondents made comments
3 of 57 comments concerned the core definition
1 of 57 suggested this was not an independent strategy but a component of other educational strategies
1 of 57 indicated that "shadow" should be further elaborated upon in the definition. ALT 1
1 of 57 indicated shadowing could apply to individuals other than clinicians. ALT 2

ALT 1:
Have clinicians directly observe other clinicians who are experts or knowledgeable in the clinical innovation.

ALT 2: "Shadow other experts"
Have individuals directly observe others who are experts or knowledgeable in the practice change/innovation.

Comments

Change physical structure and equipment

Change the physical structure and equipment (changing the layout of a room, adding equipment).

Summary:
2 of 57 respondents made comments
1 of 57 suggested clarifying the definition by specifying that the change in physical structure or equipment be tied to the innovation. See ALT 1

ALT 1:
Change the physical structure or equipment (e.g., changing the layout of a room, adding equipment) in ways that support the innovation.

Comments

Facilitate relay of clinical data to providers

Collect new clinical information from the patient/consumer and relay it to the provider outside of the traditional clinical encounter to prompt the provider to use the clinical innovation.

Ancillary Material:
Examples might include depression scores from an instrument administered in the waiting room or telephone transmission of blood pressure measurements. Existing data can be used to derive risk scores and treatment recommendations to assist the clinician in tailoring their practice. Additional examples can be found in the measurement-based care literature.

Summary:
8 of 57 respondents made comments
1 of 57 made comments on modifying the core definition
1 of 57 noted that the core definition should not emphasize "new" clinical information since there is much benefit from existing data being presented in a more useful way. See ALT 1
1 of 57 inquired whether relaying real-time data to the clinician would constitute a separate strategy
3 of 57 made comments on ancillary material

ALT 1:
Collect clinical information from the patient/consumer and their existing medical record and send it to the provider outside the traditional clinical encounter to prompt the provider to use the clinical innovation.

Comments

Use advisory boards
& workgroups

Involve multiple kinds of stakeholders in a group to oversee implementation efforts and make recommendations.

Ancillary Material:
Consider how group composition (or heterogeneity) impact stakeholder participation and take active steps to reduce response bias.

Summary:
4 of 57 respondents made comments
2 of 57 noted overlap with other stakeholder engagement focused strategies
1 of 57 suggested replacing "oversee" with "provide input and advice"
1 of 57 provided ancillary material

ALT 1:
Involve multiple kinds of stakeholders in a group to provide input and advice on implementation efforts and make recommendations.

Comments

Purposefully re-examine the implementation

Obtain commitment from stakeholders to use monitoring to adjust practice and strategies to continuously improve the implementation effort and delivery of the clinical innovation.

Summary:
4 of 57 respondents made comments
1 of 57 proposed an alternate definition ALT 1
2 of 57 felt this term overlapped with other strategies (e.g., audit and feedback, develop a formal implementation blueprint)
1 of 57 indicated that "stakeholders" was too vague and requested greater specificity

ALT 1:
Monitor progress and adjust implementation as needed.

Comments

ADDITIONAL STRATEGIES PROPOSED

The following strategies were proposed as additional discrete implementation strategies. Proposed strategies were retained if they met the criterion of actions or processes that may be used to support implementation of a given evidence-based practice or clinical innovation." Some of the proposed stra violated the "single action or process" and are not included below. Vague suggestions are noted as requiring assistance.

Promote adaptability

Identify the ways a clinical innovation can be tailored to meet local needs and clarify which elements of the innovation must be maintained to preserve fidelity.

Comments

External facilitation

A process of interactive problem-solving and support which occurs in a context of a recognized need for improvement and a supportive interpersonal relationship.

Reference: Stetler et al. Implementation Science 2006; 1:23

Comment

Identify early adopters

Identify early adopters at the local site to review their experiences with the practice innovation.

Comments

Promote network weaving

****Assistance is needed to develop a definition relating this strategy to a single action or process. It currently communicates a concept rather than a process.****

Network weaving is a disciplined approach to maximizing the benefits from naturally-occurring networks of agents. “Weavers” have the vision, the energy, and the social skills to connect to diverse individuals and groups and start information flowing to and from them.

Ancillary Material:
The weavers usually have external links outside of the community to bring in information and ideas. An example would be the nurses, doctors who staff hospitals and SNFs and the patients who rotate among these facilities. Networks are somewhat more organic than collaboratives and are often enduring and durable. See: http://www.networkweaver.com/

Comment

Provide local technical assistance
(contrasted with centralized technical assistance)

Develop and use a system to deliver technical assistance focused on implementation issues using local personnel

Ancillary Material:
For example, VA has mental health Evidence-Based Psychotherapy coordinators, Military Sexual Trauma coordinators, OEF/OIF coordinators in each facility who can provide technical assistance to other local clinicians. Local technical assistants can be connected with a broader or centralized network of technical assistants.

Comment

Name and define any proposed additional strategies here:

3. Terms where the core definition raised concerns

These are terms where some respondents indicated difficulties with the core definition such as it being too vague. The comments were not specific enough to propose an alternate definition for the strategy.

This is your opportunity to make further comments or alternate definition suggestions regarding these strategies prior to the consensus webinar where voting will occur.

If you feel an alternative definition for the strategy is warranted, propose one in the respective comment box.

If you feel the original definition for the strategy is adequate, you can leave the respective comment box empty.

Ancillary material contributions are welcome but not necessary.

****Reminder. Many Round 1 comments noted the similarity or overlap of many of the strategies. Concept Mapping data from a later phase in this study will be used to provide a quantitative basis for considering the merging of terms.

Assess for readiness
and identify barriers

Assess various aspects of an organization to determine its degree of readiness to implement, barriers that may impede implementation, and strengths that can be used in the implementation effort.

Ancillary Material:
The assessment may focus on agency finances, staffing levels, other material or logistical resources needed or available to support implementation, leadership support, the organizational priority for change, the presence of successful experience with QI techniques and change management, other services provided, community support, stakeholder attitudes, beliefs and perceptions of evidence for the innovation/change, rationale for current practices, organizational climate and culture, structure, and decision-making styles, the perceived needs of frontline stakeholders to implement the change/innovation (consider adaptation needs and limits). The readiness assessment can be used to vet, eliminate, or prioritize implementation sites. Some barriers can be difficult to observe prior to implementation. There are also specific measures created to assess readiness to change that could be helpful (e.g., Lehman, Greener, & Simpson, 2002; Weiner, Amick, & Lee, 2008).

Summary:
18 of 57 respondents made comments.
8 of 57 noted substantial overlap with "conduct local needs assessment"
3 of 57 suggested splitting the core definition by specifying separate terms for assessing readiness and for identifying barriers
9 of 57 respondents made additional ancillary material contributions

Comments

Stage
implementation
scale up

Phase implementation efforts by starting with small pilots or demonstration projects and gradually moving to system-wide rollout.

Ancillary Materials:
This involves an iterative process that often results in adaptations. Strategies for integrating pilot feedback into the scale-up/spread process should be established in advanced. Depending on the innovation, piloting may also involve phasing in elements/components of the practice change. Many innovations involve more than one service (e.g., inpatient and outpatient; primary care and specialty care) and the scaling-up/spread process may have different needs to address the interactions among services (e.g. needs related to ensure continuity of care while connecting services).

Summary:
10 of 57 respondents made comments
2 of 57 respondents made comments regarding the label for the strategy
1 of 57 indicated the strategy label was confusing
1 of 57 indicated that "scale-up and spread" is more commonly used
1 of 57 comments concerned the core definition noting that the term seemed to be more of a general principle than a strategy
4 of 57 respondents made comments regarding ancillary materials

Comments

Model and simulate
change

Model or simulate the change that will be implemented prior to implementation.

Ancillary Materials:
These efforts could involve computer simulations, walkthrough simulation exercises, or modeling the potential overall impact of stakeholder's behavior change. This approach is often more relevant for complex multi-component innovations.

Summary:
10 of 57 respondents made comments
1 of 57 made comments regarding the core definition suggesting that "change" be changed to "innovation"
2 of 57 made comments regarding ancillary information indicating ancillary material should better specify the conditions where modeling and simulating change would be appropriate.

Comments

Mandate change

Declare that the innovation will be implemented.

Ancillary Material:
Implementers often do not have the power to mandate change themselves. There is often the need to work with organizational leadership to develop buy-in and lobby for a change mandate

Summary:
7 of 57 respondents made comments
2 of 57 respondents made comments involving the core definition
1 of 57 questioned whether this term was redundant with "involve governing boards"
1 of 57 suggested that the authority of the mandate be clarified
2 of 57 provided ancillary material

Comments

Develop effective educational materials

Develop and format guidelines, manuals, toolkits and other supporting materials in ways that make it easier for stakeholders to learn about the innovation and for clinicians to learn how to deliver the clinical innovation.

Ancillary Material:
Create eye-catching, easy-to-use documents. Distill complex information into easier-to-learn components. Consider teaching skills modularly. Use different forms of media. Target messages for different audiences. Educational materials should reflect principles of adult learning theory. It is important to assess whether the technology infrastructure can accommodate educational media (e.g., firewalls, old hardware, old software). Consider how the materials will be used over time to train new or rotating staff, refresh staff knowledge, or be incorporated into existing supervision, competency, and performance review structures. The REP framework provides relevant suggestions under its "packaging" domain. Formative evaluation feedback is often used to refine educational materials.

Summary:
11 of 57 respondents made comments
2 of 57 noted that "effective" in the label presumes that educational materials have all been tested for effectiveness while this is often not the case
6 of 57 comments focused on ancillary information

Comments

Develop a glossary of
implementation

Develop a glossary to promote common understanding about implementation among the different stakeholders.

Ancillary Material:
When compiling a glossary reflect as to whether the terms being introduced are essential.

Summary:
11 of 57 respondents made comments
7 of 57 made comments on the core definition
3 of 57 indicated that the core definition was too vague
3 of 57 felt this term was redundant with "develop effective educational materials"
1 of 57 felt this term was redundant with "develop a formal implementation blueprint"
1 of 57 provided ancillary material

Comments

Conduct ongoing
training

Plan for and conduct training in the clinical innovation in an ongoing way.

Ancillary Materials:
This can include follow-up training, advanced training, booster training, purposefully spaced training, training to competence, integration of off the- job and on-the-job training, structured supervision, the introduction of concepts in a specific sequence to ensure mastery, and trainings based on the level of clinician knowledge. Trainings can be in-person, on the web, or technology-assisted. Ongoing training may focus on individuals or involve groups.

Summary:
7 of 57 Respondents made comments
1 of 57 suggested splitting the core definition to differentiate training primarily delivered by another human being from primarily technology-delivered training
2 of 57 comments involved ancillary materials

Comments

Make training
dynamic

Vary the information delivery methods to cater to different learning styles and work contexts, and shape the training in the innovation to be interactive.

Ancillary Material:
This includes efforts to divide material into small time intervals and the use of small group breakouts, audience response systems, and other measures. The interactive component of training can be very dynamic with participants actively contributing to the training content and engaging in problem solving and identifying solutions that can be tested.

Summary:
11 of 57 respondents made comments
3 of 57 comments focused on the core definition
2 of 17 indicated there did not need to be a distinction between "conduct ongoing training" and making training dynamic where the latter would be ancillary to the former
1 of 17 indicated this term combines two issues that could be separate: tailoring and interactive/active learning strategies
2 of 17 provided ancillary material

Comments

Conduct educational
outreach visits

Use a trained person who meets with providers in their practice settings to educate providers about the clinical innovation with the intent of changing the provider’s practice.

Ancillary Material:
Consider using well respected models for these visits. Some initiatives may require regular educational outreach as part of maintaining the innovation/practice change.

Summary:
4 of 57 respondents made comments
2 of 57 comments concerned the core definition
1 of 57 suggested removing "academic detailing" as an example synonym from the ancillary materials as academic detailing is not a discrete implementation strategy. This ancillary material has been removed.
1 of 57 noted concern that "intent of changing the provider's practice" may lead some to believe this strategy entails more than providing information (e.g., behavioral rehearsal)
2 of 57 respondents contributed ancillary material.

Comments

Use mass media

Use media to reach large numbers of people to spread the word about the clinical innovation.

Ancillary Materials:
Mass media may include television, newspapers, magazines, radio, electronic social media, listserves, mass email campaigns, mass mailings, robocalls as methods for spreading information. Targets of the media campaign may be clinicians, potential consumers of the innovation, or their associates.

Summary:
7 of 57 respondents made comments
1 of 57 noted the definition was too vague
4 of 57 made ancillary contributions

Comments

Prepare patients/
consumers to be
active participants

Prepare patients/consumers to be active in their care, to ask questions, and specifically to inquire about care guidelines, the evidence behind clinical decisions, or about available evidence-supported treatments.

Ancillary Material:
Asking questions can also involve preparing consumers to inquire about specific practices.

Summary:
4 of 57 respondents made comments
2 of 57 had concern with the core definition
1 of 57 inquired whether the definition should only focus on patients (i.e., involve other stakeholders)
1 of 57 noted difficulty distinguishing this strategy from "Involve patients/consumers and family members"
1 of 57 made ancillary material contributions

Comments

Penalize

Penalize providers financially for failure to implement or use the clinical innovation.

Summary:
4 of 57 respondents made comments
1 of 57 noted that the label "penalize" seems odd relative to the other terms

Comments

Create new clinical
teams

Change who serves on the clinical team, adding different disciplines and different skills to make it more likely that the clinical innovation is delivered or more successful.

Summary:
5 of 57 respondents made comments
2 of 57 noted similarity between this term and "revise professional roles"
1 of 57 noted that the title suggests creating new teams (e.g., in a system not using team-based care approaches thus far); the description addresses changes in (existing) teams

Comments

Start a purveyor
organization

Start a separate organization that is responsible for disseminating the clinical innovation. It could be a for-profit or non-profit organization.

Ancillary Material:
This strategy can address concerns (e.g., conflict of interest) for situations where it is desirable to have fidelity monitors that are independent from the care setting.
This could be a for-profit or nonprofit organization. It could be "licensed" by a university if the innovation was born within an academic setting.

Summary:
7 of 57 made comments
3 of 57 felt "purveyor organization" was an obscure turn of phrase
1 of 57 provided ancillary material

Comments

Develop tools for
quality monitoring

Develop, test, and introduce into quality-monitoring systems the right input – the appropriate language, protocols, algorithms, standards, and measures (of processes, patient/consumer outcomes, and implementation outcomes) that are often specific to the innovation being implemented.

Ancillary Material:
These tools should be flexible enough to reflect fidelity even after adaptations to the setting or client. The performance sites can benefit when these tools are available locally. Measurement tools can be coordinated with other strategies to encourage or reward performance that is in alignment with the clinical innovation. See Krein et al., (2008) for an example of this process.

Summary:
6 of 57 respondents made comments
2 of 57 indicated overlap between this term and "develop and organize quality monitoring systems)
1 of 57 noted the label is misleading because the definition covers "Develop and implement" tools for quality monitoring
2 of 57 contributed ancillary material

Comments

Audit and provide feedback

Collect and summarize clinical performance data over a specified time period and give it to clinicians and administrators in the hopes of changing provider behavior.

Ancillary Material:
The information may have been obtained from a variety of sources, including medical records, computerized databases, observation, or feedback from patients. A performance evaluation could also be considered as audit and feedback if it included specific information on clinical performance. The feedback summary may include recommendations. Some feedback can be designed to guide a clinician in improving fidelity. Performance data may include process variables, outcomes, or fidelity measures. Feedback can include mandatory performance measures that are related to benchmarks from the literature or normative data within an organization or industry. Provider behavior change can be an index of the impact of organizational change strategies separate from strategies targeting the clinician directly.

Summary:
6 of 57 respondents made comments
1 of 57 indicated audit and feedback are two separate strategies
5 of 57 made ancillary material contributions

Comments

Use data warehousing techniques

Integrate clinical records across facilities and organizations in order to facilitate implementation across systems.

Ancillary Material:
Records that include variables that can serve as outcome measures are particularly useful.

Summary:
6 of 57 respondents made comments
3 of 57 found the label or the definition vague
1 of 57 noted overlap with "Change record systems"
1 of 57 provided ancillary material

Comments

Use an improvement/ implementation advisor

Seek guidance from experts in implementation.

Ancillary Material:
This could include consultation with outside experts such as university-affiliated faculty members, or hiring quality improvement experts or implementation professionals.

Summary:
3 of 57 respondents made comments
1 of 57 suggested changing the label noting that "Improvement Advisor" has a specific role in Quality Improvement methodology a) developing curriculum and tools for the learning collaborative and b) coaching individuals and teams.

Comments

Change accreditation or membership requirements

Strive to alter accreditation standards so that they require or encourage use of the clinical innovation. Work to alter membership organization requirements so that those who want to affiliate with the organization are encouraged or required to use the clinical innovation.

Summary:
3 of 57 respondents made comments
1 of 57 suggested developing separate terms for provider and hospital/organization level accreditation/membership requirements

Comments

4. Terms with no comments about the core definition or ancillary materials.

If respondents commented on these strategies in Round 1, the comments were not clearly about changing the core definition or the addition of ancillary material.

This is your opportunity to make comments or alternate definition suggestions regarding these strategies prior to the consensus webinar where voting will occur.

If you feel an alternative definition for the strategy is warranted, propose one in the respective comment box.

If you feel the original definition for the strategy is adequate, you can leave the respective comment box empty.

Ancillary material contributions are welcome but not necessary. Part 2 of the study will provide opportunities to contribute ancillary material for the strategies in the context of particular implementation initiatives.

****Reminder. Many Round 1 comments noted the similarity or overlap of many of the strategies. Concept Mapping data from a later phase in this study will be used to provide a quantitative basis for considering the merging of terms.

Distribute educational materials

Distribute educational materials (including guidelines, manuals and toolkits) in person, by mail, and/or electronically.

Summary:
5 of 57 respondents made comments
3 of 57 comments concerned the core definition, all indicated that this term could be merged with "Develop effective educational materials". Combining terms will be addressed in the Concept Mapping activity.

Comments

Reduce or increase patient/consumer fees

Create fee structures where patients/consumers pay less for preferred treatments (the clinical innovation) and more for less preferred treatments.

Summary:
4 of 57 respondents made comments
0 of 57 suggested changes to the core definition or provided ancillary material

Comments

Place on fee for
service lists/
formularies

Work to place the clinical innovation on lists of actions for which providers can be reimbursed (e.g., a drug is placed on a formulary, a procedure is now reimbursable).

Summary:
3 of 57 respondents made comments
1 of 57 indicated this term overlapped with other financial strategies

Comments

Fund and contract for
the clinical
innovation

[Governments and other payers of services] issue requests for proposals to deliver the innovation, use contracting processes to motivate providers to deliver the clinical innovation, and develop new funding formulas that make it more likely that providers will deliver the innovation.

Summary:
4 of 57 respondents made comments
1 of 57 indicated this term overlapped with other financial strategies

Comments

Revise professional roles

Shift and revise roles among professionals who provide care and redesign job characteristics.

Ancillary Material
This includes the expansion of roles to cover provision of the clinical innovation and the elimination of service barriers to care, including personnel policies.

Summary:
4 of 57 respondents made comments
0 of 57 made specific suggestions for the core definition or ancillary materials

Comments

Centralize technical assistance

Develop and use a system to deliver technical assistance focused on implementation issues.

Ancillary Material:
This could be the designation of a lead technical assistance organization (could also be responsible for training). The lead technical assistance entity can develop other mechanisms (e.g., call-in lines or websites) in order to share information on how to best implement the clinical innovation.

Summary:
4 of 57 respondents made comments
0 of 57 respondents suggested changes to the core definition or ancillary material

Comments

Intervene with patients/ consumers to enhance uptake and adherence

Intervene with patients/consumers to increase uptake of and adherence to clinical treatments.

Ancillary Material:
This includes consumer/patient reminders and financial incentives to attend appointments.

Summary:
7 of 57 respondents made comments
3 of 57 noted overlap of this term with others related to education or stakeholder engagement

Comments

Organize clinician
implementation
team meetings

Develop and support teams of clinicians who are implementing the innovation and give them protected time to reflect on the implementation effort, share lessons learned, and support one another's learning.

Summary:
5 of 57 respondents made comments
1 of 57 noted overlap with "Revise professional roles" and "create new clinical teams"

Comments

Create or change
credentialing and/or
licensure standards

Create an organization that certifies clinicians in the innovation or encourages an existing organization to do so. Change governmental professional certification or licensure requirements to include delivering the innovation. Work to alter continuing education requirements to shape professional practice toward the innovation.

Summary:
2 of 57 respondents made comments
0 of 57 made comments on the core definition or provided ancillary materials

Comments

5. Terms with few comments on ancillary materials.

Respondents indicated no changes to the core definitions of these strategies. A few respondents (<3) did contribute ancillary materials.

This is your opportunity to make further comments or alternate definition suggestions regarding these strategies prior to the consensus webinar where voting will occur.

If you feel an alternative definition for the strategy is warranted, propose one in the respective comment box.

If you feel the original definition for the strategy is adequate, you can leave the respective comment box empty.

Ancillary material contributions are welcome but not necessary. Part 2 of the study will provide opportunities to contribute ancillary material for the strategies in the context of particular implementation initiatives.

****Reminder. Many Round 1 comments noted the similarity or overlap of many of the strategies. Concept Mapping data from a later phase in this study will be used to provide a quantitative basis for considering the merging of terms.

Build a coalition

Recruit and cultivate relationships with partners in the implementation effort.

Ancillary Material:
Partnerships can develop around cost-sharing, shared resources, shared training, and the division of responsibilities among partners. This work may proceed naturally from local consensus discussions. Coalition members commonly have defined roles in the implementation effort.

Summary:
9 of 57 respondents made comments
5 of 57 respondents made comments involving the core definition noting that this term was duplicative of other terms and lacked detail.
1 of 57 contributed ancillary material

Comments

Develop resource
sharing agreements

Develop partnerships with organizations that have resources needed to implement the innovation.

Ancillary Material:
As an example, a group of providers could strike a relationship with a microbiology lab to conduct specialized lab work needed to implement an innovation efficiently.

1 of 57 respondents made comments
1 of 57 indicated that the example provided in the ancillary material was of poor quality

Comments

Conduct educational
meetings

Hold meetings targeted toward providers, administrators, other organizational stakeholders, and community, patient/consumer, and family stakeholders to teach them about the clinical innovation.

Ancillary Material:
The content of the education may include information regarding what to expect as implementation moves forward. It is often useful to have recordings or other materials from the educational meetings available to those who cannot attend the meetings (e.g., those covering patient care at the time of the meeting, new hires subsequent to the meeting).

Summary:
6 of 57 respondents made comments
1 of 57 concerned the core definition indicating this term was redundant with "Training" strategies
2 of 57 contributed ancillary material.

Comments

Inform local opinion
leaders

Inform providers identified by colleagues as opinion leaders or “educationally influential” about the clinical innovation in the hopes that they will influence colleagues to adopt it.

Ancillary Material
The opinions of individuals who refer people to services, or who initiate the connection to services also serve a key opinion role.
It can also be important to ensure that opinion leaders do not serve as implementation obstacles if they are not actively promoting the innovation.

Summary:
6 of 57 respondents made comments
3 of 17 noted concerns with the core definition indicating this term may be redundant with other terms particularly "identifying and preparing champions"
2 of 17 made ancillary contributions

Comments

Increase demand

Attempt to influence the market for the clinical innovation to increase competition intensity and to increase the maturity of the market for the clinical innovation.

Summary:
4 of 57 respondents made comments
0 of 4 comments concerned the core definition
2 of 4 comments requested more ancillary material to provide guidance in enacting this strategy

Comments

Work with educational institutions

Encourage educational institutions to train clinicians in the innovation.

Ancillary Material:
This strategy fits well with innovations requiring clinical training and skill.

Summary:
10 of 17 made comments
7 of 17 indicated this term seemed duplicative with "develop academic partnerships"
1 of 17 contributed ancillary material

Comments

Use capitated
payments

Pay providers a set amount per patient/consumer for delivering clinical care.

Ancillary Material:
This is an implementation strategy to the degree that it frees the clinician to provide services that they may have been disincentivized to provide under a fee-for-service structure. This may be helpful to motivate clinicians to use certain clinical innovations. These changes often come about as part of policy changes that alter fee structures, alter coverage, or add items to reimbursement formularies.

Summary:
4 of 57 respondents made comments
1 of 57 suggested ancillary material

Comments

Use other payment
schemes

Introduce such payment approaches (in a catch-all category).

Ancillary Material:
These approaches may involve prepayment and prospective payment for service, provider salaried service, the alignment of payment rates with the attainment of patient/consumer outcomes, and the removal or alteration of billing limits (such as numbers of encounters that are reimbursable). Payment may also be based on measures of treatment fidelity. These are implementation strategies to the degree that they free the clinician to provide the clinical innovation. Others motivate the clinician to provide better service.

Summary:
4 of 57 respondents made comments
2 of 57 indicated this strategy did not appear unique from other financial strategies on the list
1 of 57 made ancillary material contributions

Comments

Access new funding

Access new or existing money to facilitate the implementation.

Ancillary Material:
This could involve new uses of existing money, accessing block grants, shifting funding from one program to another, cost-sharing, passing new taxes, raising private funds, or applying for grants. The money may be used to fund the delivery of a clinical innovation or to support other time limited actions needed for initial implementation (e.g., purchase material or logistical support, training).

Summary:
4 of 57 respondents made comments
1 of 57 indicated this term overlapped with other financial strategies
1 of 57 contributed ancillary material

Comments

Make billing easier

Make it easier to bill for the clinical innovation.

Ancillary Material:
This might involve requiring less documentation, “block” funding for delivering the innovation, and creating new billing codes for the innovation. Developing progress note templates to facilitate documentation of the clinical innovation can also decrease the burden for obtaining payment.

Summary:
3 of 57 respondents made comments
1 of 57 contributed ancillary materials

Comments

Change service sites

Change the location of clinical service sites to increase access.

Ancillary Material:
This can include collocating different services to better implement complex clinical innovations that require multiple disciplines or services, telemedicine, or bringing the services to the client in the community or other clinically relevant setting (e.g., busy public spaces for a client with PTSD).

Summary:
3 of 57 respondents made comments
2 of 57 provided ancillary material

Comments

Change records
systems

Change records systems to allow better assessment of implementation or of outcomes of the implementation.

Ancillary Material:
These changes my include modifying the format of progress notes and treatment plans to reflect the innovation (evidence-based practice) being implemented.

Summary:
2 of 57 respondents made comments
1 of 57 provided ancillary material

Comments

Develop and
organize quality
monitoring systems

Develop and organize systems and procedures that monitor clinical processes and/or outcomes for the purpose of quality assurance and improvement.

Ancillary Material:
This includes developing systems for monitoring through peer reviews, collecting data from patients/consumers, clinicians, and supervisors, and using administrative and electronic record data. This category of strategies also includes the design of disease-specific clinical registries, where clinical information and tools (graphical representations, real-time report cards, comparisons with benchmarks, etc.) are available to care team members. These systems may inform audit and feedback strategies. Some intensive fidelity monitoring activities (e.g., psychotherapy recordings) are more practical at random, but not infrequent, intervals.

Summary:
4 of 57 respondents made comments
1 of 57 noted overlap with "audit and feedback"
1 of 57 provided ancillary material

Comments

Remind clinicians

Develop reminder systems designed to prompt clinicians to recall information or use the clinical innovation.

Ancillary Material:
The reminder could be patient or encounter specific, provided verbally, on paper, or on a computer screen. Reminders may also be organized temporally (prior to service, during service, or following service delivery). Computer-aided decision support and drug dosages are included in this strategy.

Summary:
4 of 57 respondents made comments
2 of 57 contributed ancillary material

Comments

Obtain and use
patient/
consumer and family

feedback

Use mechanisms to increase patient/consumer and family feedback on the implementation effort.

Ancillary Material:
This could include complaint forms or methods to funnel feedback to change managers or advisory boards. Consider whether anonymous feedback formats are appropriate.

Summary:
4 of 57 respondents made comments
1 of 57 noted overlap with other stakeholder engagement strategies
2 of 57 provided ancillary material

Comments

Provide clinical
supervision

Provide clinicians with ongoing supervision. Provide training for clinical supervisors who will supervise clinicians who provide the innovation.

Ancillary Material:
Supervisor training often needs to include training in how to supervise the innovation.
See Nadeem, Gleacher, and Beidas (2013) for a discussion of the distinction between consultation and supervision.

Summary:
6 of 57 respondents made comments
3 of 57 noted poor differentiation of this term from providing ongoing training or consultation
2 of 57 made ancillary material contributions

Comments

Conduct cyclical
small tests of change

Implement changes in a cyclical fashion using small tests of change before taking changes system-wide. Results of the tests of change are studied for insights on how to do better. This process continues serially over time and refinement is added with each cycle.

Ancillary Material:
Tests of change benefit from systematic measurement. Two common small tests of change cycling strategies are "Plan-Do-Study-Act" from Deming's quality management work and six sigma's Define-Measure-Analyze-Improve-Control sequence.

Summary:
3 of 57 respondents made comments
2 of 57 noted overlap with other strategies (i.e., stage implementation scale-up, purposefully reexamine implementation)
1 of 57 provided ancillary material

Comments

Use data experts

Involve, hire and/or consult experts in data management to shape use of the considerable data that implementation efforts can generate.

Ancillary Material:
Consider engaging data experts early in the implementation planning process.

Summary:
2 of 57 respondents made comments
1 of 57 contributed ancillary material

Comments

Capture and share
local knowledge

Capture local knowledge from implementation sites on how implementers and clinicians made something work in their setting and then share it with other sites.

Ancillary Materials:
This strategy is often coordinated with centralized technical assistance and learning collaboratives. There are multiple techniques for capturing local knowledge. Example techniques can be found at www.liberatingstructures.com.

Summary:
4 of 57 respondents made comments
2 of 57 comments felt there was overlap with other terms (e.g., learning collaboratives and centralize technical assistance)
1 of 57 provided ancillary material

Comments

Change liability laws

Participate in liability reform efforts that make clinicians more willing to deliver the clinical innovation.

Ancillary Material:
Liability reform can also make clinicians less willing to deliver alternatives to the clinical innovation.

Summary:
4 of 57 respondents made comments
1 of 57 contributed ancillary material

Comments

6. Terms with many comments on ancillary materials.

Respondents indicated no changes to the core definitions of these strategies. Three or more respondents did contribute ancillary materials.

This is your opportunity to make further comments or alternate definition suggestions regarding these strategies prior to the consensus webinar where voting will occur.

If you feel an alternative definition for the strategy is warranted, propose one in the respective comment box.

If you feel the original definition for the strategy is adequate, you can leave the respective comment box empty.

Ancillary material contributions are welcome but not necessary. Part 2 of the study will provide opportunities to contribute ancillary material for the strategies in the context of particular implementation initiatives.

****Reminder. Many Round 1 comments noted the similarity or overlap of many of the strategies. Concept Mapping data from a later phase in this study will be used to provide a quantitative basis for considering the merging of terms.

Conduct local needs assessment

Collect and analyze data related to the need for the innovation.

Ancillary Materials:
This assessment could be focused on:
+ outcomes of usual care
+ the process of care
+ the description of usual care and its distance from the evidence based care (e.g., gaps in care)
+ opinions from stakeholders (including patients) on (a) the needs for any innovation (i.e., tension for change), (b) the need for a specific innovation, or (c) the special considerations for delivering the innovation in the local context.
Common methods include surveys, focus groups, key informant interviews, direct observation, and data mining administrative records for identifying target populations and identifying baseline care process and outcome clinical care data. If the change involves multiple sites/facilitates then examine practice variation across facilities and outline strategies for the needs assessment to support a standardized approach across sites. Randomization strategies may be necessary to reduce response bias.

Summary:
20 of 57 respondents made comments.
0 of 57 respondents suggested a change in the core definition.
3 of 57 respondents noted substantial overlap between this term and "Assess for Readiness & Identify Barriers"
11 of 57 respondents made comments about refining/expanding material ancillary to the core definition. Suggestions have been incorporated into ancillary material.

Comments

Visit other sites

Visit sites where a similar implementation effort has been considered successful.

Ancillary material:
Clarifying the goals of the site visit prior to making the visit is particularly useful. Compare and contrast the features of your site with the comparison site in preparation for the visit to better inform the visit objectives. Identify adaptations made in implementing the innovation and any perceived impact on the effectiveness of the innovation/practice change. Document facilitators and lessons learned. Much can be learned from visiting sites that have a strong track record for successfully implementing a wide variety of other innovations/practice changes. Consulting with sites where implementation has stalled or failed can also provide useful information. Sites also benefit from sharing implementation planning and execution notes virtually (i.e., information exchange is not limited to physical visits).

Summary:
16 of 57 respondents made comments
0 of 57 made suggestions regarding changing the core definition
9 of 57 comments focused on ancillary material

Comments

Conduct local
consensus
discussions

Include providers and other stakeholders in discussions that address whether the chosen problem is important and whether the clinical innovation to address it is appropriate.

Ancillary Material:
Each project will want to identify the stakeholders relevant to their project. Each project will need to identify whether the goal of the consensus discussion is to characterize consensus or build consensus. Utilizing community based participatory research principles may be relevant to many innovations.

Summary:
12 of 57 respondents made comments
3 of 57 commented on the core definition indicating that they viewed this as a component of a needs assessment.
3 of 57 respondents made comments regarding ancillary content:

Comments

Involve executive
boards

Involve existing governing structures (e.g., boards of directors, medical staff boards of governance) in the implementation effort, including the review of data on implementation processes.

Ancillary Material:
For settings that do not have a governing board, other types of leadership with "top-down" powers may be involved. Examples include administrative leadership, clinical leadership, policy makers, insurance providers or other payment systems.

Summary:
9 of 57 respondents made comments
5 of 57 commented on the core definition
4 of 57 suggested including other divisions of leadership or clarifying what level of leadership this applies to. Other divisions suggested have been included in the ancillary material.
1 of 57 suggested collapsing this strategy with "conduct local consensus discussions" as both involve obtaining stakeholder feedback

Comments

Recruit, designate,
and train for
leadership

Recruit, designate, and train leaders for the change effort.

Ancillary Materials:
Change efforts require certain types of leaders, and organizations may need to recruit accordingly, rather than assuming that their current personnel can implement the change. Designated change leaders can include an executive sponsor and a day-to-day manager of the effort. Change leaders should consider how to establish effective supervisory lines for clinical practice innovations that are enacted by clinicians when the change leader does not have similar clinical responsibilities.

Summary:
9 of 57 respondents made comments
4 of 57 indicated poor differentiation from "Identify and prepare Champions" or that these two terms may both be members of the same category
3 of 57 made comments regarding ancillary material

Comments

Obtain formal
commitments

Obtain written commitments from key partners that state what they will do to implement the innovation.

Ancillary Material:
Formal commitments should clarify roles, responsibilities, and detail tangible and non-tangible benefits (e.g., Wells & Jones community partnership work). Ensure that key roles are supported within the organization (e.g., workload release credit for providing and receiving supervision in a new clinical practice)

Summary:
5 of 57 respondents made comments
5 of 57 commented on ancillary material

Comments

Develop academic
partnerships

Partner with a university or academic unit for the purposes of shared training and bringing research skills to an implementation project.

Ancillary Material:
It is common to encounter HIPAA and other legal limitations to academic partnerships. In some instances formal relationships (e.g., contracts, MOUs) will be required. Not all academics have a full understanding of practice level stakeholder needs and this should be considered while developing this partnership. In settings where "research" is a dirty word, evaluation may be a more useful way to characterize the activity.

Summary:
5 of 57 respondents made comments
1 of 57 indicated that the term seems to be a member of a broader "involve stakeholders" category.
4 of 57 commented on ancillary material

Comments

Use train-the-trainer
strategies

Train designated clinicians or organizations to train others in the clinical innovation.

Ancillary Material:
Restrictions regarding who can serve as a trainer is idiosyncratic to the innovation/practice change and these restrictions should be explored in the planning phase.

Summary:
3 of 57 respondents made comments
3 of 57 indicated that the ancillary material was awkwardly worded--it has been revised.

Comments

Alter incentive/
allowance structures

Work to incent the adoption and implementation of the clinical innovation.

Ancillary Materials:
Incentives may be based on the performance of individual clinicians or larger performance units at the organizational level.
The incentive could be in the form of an increased rate of pay to cover the incremental costs associated with implementing the clinical innovation. The incentive could be through loan reduction/forgiveness to clinicians as an incentive to learn an innovation. This category of financial strategies also includes the elimination of any perverse incentives (incentives that become a barrier to receiving appropriate care). An incentive suggests the payment is tied to performing the clinical action or improving outcomes. An allowance suggests that the clinician/organization is not required to perform the clinical action or meet the performance standard.

Summary:
5 of 57 respondents made comments
3 of 57 comments concerned ancillary material

Comments
