# Supplementary material for: A refined compilation of implementation strategies: results from the Expert Recommendations for Implementing Change (ERIC) project
Source: Implement Sci. 2015 Feb 12;10:21. doi: 10.1186/s13012-015-0209-1 (PMC4328074; doi:10.1186/s13012-015-0209-1)
Supplement: Additional file 3: — Expert Recommendations for Implementing Change (ERIC) voting guide. This voting guide was mailed to participants prior to modified Delphi Round 3. [file 13012_2015_209_MOESM3_ESM.docx]

**Additional File 3:**

Expert Recommendations for Implementing Change (ERIC)

Voting Guide

In this webinar you will be voting on suggested modifications to the discrete implementation strategies included in the ERIC project. We had a high level of participation in the modified Delphi surveys that preceded this webinar and as a result will have 26 strategies to vote on. Many of these votes may be easy decisions, while others will be more difficult. To support the voting process a separate **“ERIC Voting Notes” file** has been provided that presents the label for the strategy and all proposed definitions for voting. It also contains columns where you can record your vote in advance of the meeting. This is for your personal use to help keep the voting process. We want to be able to efficiently record consensus for strategies where consensus comes easy so that there will be adequate time to discuss terms with low consensus. **Please print out this file and have your votes for the approval poll prepared before entering the webinar.**

The webinar will last AT LEAST 60 Minutes and if possible, please allow up to 90 minutes in your schedule in case discussion results in and extended webinar. The invitation to the webinar will include the link to register yourself into the program and will provide you with the phone number for the accompanying conference call. You will need to dial in to the conference call to hear the audio for the webinar. Please mute your phone to provide your fellow expert panelists a distraction free listening experience. **It often takes a few minutes to enter the webinar. If possible try to enter the webinar 5 minutes in advance. We will start on time.**


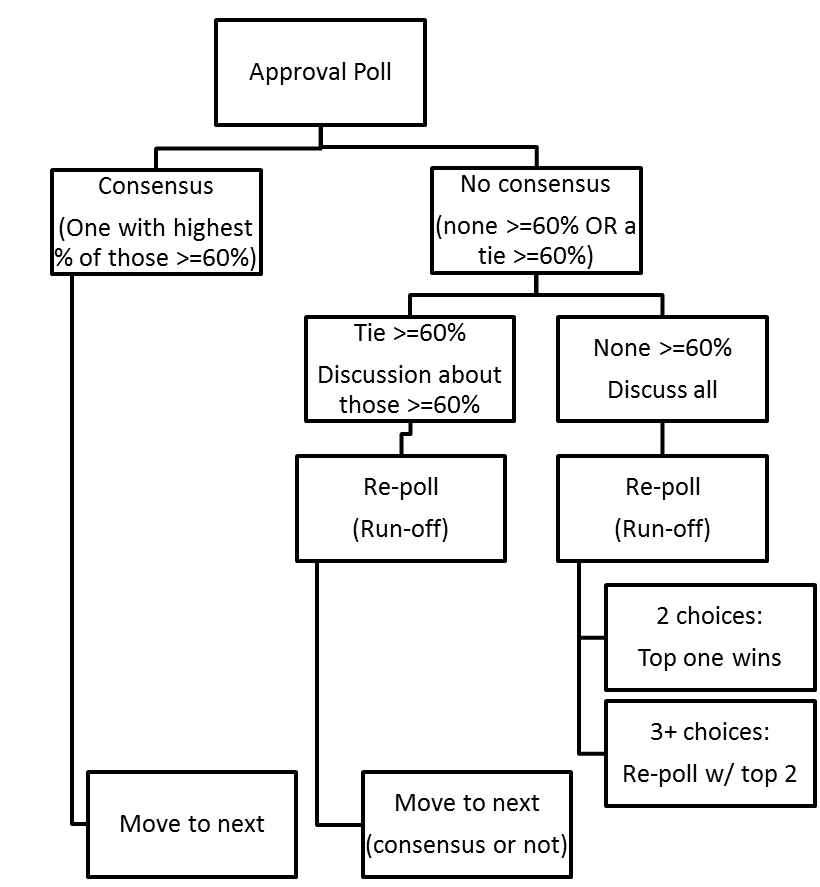
What is the Focus of the Voting Process?

Voting focuses on characterizing consensus regarding the definition of discrete implementation strategies. Discrete implementation strategies are defined as single actions or processes that may be used to support implementation of a given evidence-based practice or clinical innovation^[[1]](#footnote-1)^. Powell et al. (2012) served as the source for the 68 initial strategies included in the ERIC project, and 5 additional strategies were proposed during the earlier modified Delphi survey rounds preceding this webinar. 21 of the 68 original strategies received comments that resulted in the proposal of alternative definitions. Only strategies with alternative definitions and the 5 proposed strategies are included in the voting process (total = 26). The figure to the right provides an overview of the voting process.

What are the Specifics of the Voting Process?

The webinar will utilize online polling to characterize consensus for the strategies’ definitions.

- Approval Polling: in approval polling you are free to endorse ALL of the definitions you find acceptable for a strategy^[[2]](#footnote-2)^. The first poll for each strategy will be an approval poll.
  - If one definition receives ≥60% approval (and is not tied with another) then that definition is declared the winner and will be retained for the rest of the ERIC process.
  - If there is no ≥60% winner in the approval poll, there will be a discussion period (see below) followed by runoff polling.
- Runoff polling: in runoff polling you can only endorse one voting alternative.
  - If there are only two choices, then the definition receiving the most votes will be retained for the rest of the ERIC process.^[[3]](#footnote-3)^
  - If there are three or more choices, there will be two rounds.
    - Round 1 identifies the top two definitions for the strategy
    - Round 2: the definition receiving the most votes will be retained for the rest of the ERIC process.

How are Discussions Structured?

Discussions only occur after an approval poll fails to identify a clear winner with ≥60% approval or a tie. If a tie involves definitions with ≥60% approval, then only those definitions ≥60% are open for discussion. Discussions are limited to 5 minutes total for each strategy due to time limitations. The moderator will ask panelists interested in contributing to the discussion to “raise their hand” in the webinar. You raise your hand by clicking on the hand button on the webinar control panel. When your hand is raised the icon will change from an upward pointing green arrow to a downward pointing red arrow (multiple clicks raise or lower your hand). When you are called upon to comment, unmute your phone and try to limit your comments to 60 seconds or less to provide adequate time for other participants to make comments. If someone makes a comment that is similar to the one you planned on making, then please consider “un-raising” your hand by clicking on the hand icon again. When there are no additional hands raised or the 5 minute discussion time has expired, the webinar will return to runoff voting as described above. Note, given the structured voting process used in this project, the question box will not be monitored or used, even though it will be visible to you.


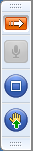

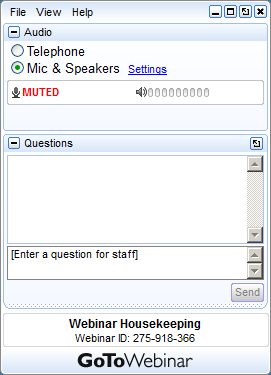


- Constructive discussion is anticipated to focus on the relative merits of the proposed definitional alternatives or the needs of an adequate definition.
- Remember: issues of related evidence and practicality/feasibility are beyond the scope of obtaining consensus on the definitions. Wordsmithing or editing for clarity of speech or grammar can be suggested via an email as long as the suggestion does not substantively change the meaning of the definition.
- Ancillary materials: From the Powell et al. (2012) paper we separated definitions from ancillary material that was descriptive or supportive of the definition in some manner. Some of feedback that we received in the modified Delphi surveys will be included in ancillary material rather than in the definition and therefor may not appear in the alternative definitions.

How was Vote-able Material Determined?

ERIC investigative team members coded participant comments from the earlier modified Delphi survey rounds as either a) concerning the core definition, b) concerning material ancillary to the definition, c) editorial comments.

1. Some core definition comments included a complete proposal for an alternative definition while some described concerns or minor modifications. Suggestions that improved the grammar or readability of the definition were adopted based on investigative team consensus that the change did not alter the core meaning of the definition. When the investigative team had concerns that minor modifications may significantly change the meaning or emphasis of a strategy, an alternative definition was constructed to present the change to expert panel members.
2. Ancillary material is intended to be more fluid and accommodate additional guidance and examples regarding enacting the strategy. Many comments fell under this category and we expect to aggregate additional ancillary material in later stages of the ERIC process.
3. Editorial comments ranged from endorsements of an original definition or a particular alternative to comments regarding the evidence base (or lack thereof) for a strategy. The ERIC process is not designed to evaluate the evidence base for the strategies included, but we will analyze this data this at a later date.

1. Powell, B.J., McMillen, J.C., Proctor, E.K., Carpenter, R.C., Griffey, R.T., Bunger, A.C., Glass, J.E., & York, J.L. (2012). A compilation of strategies for implementing clinical innovations in health and mental health. *Medical Care Research and Review*, 69, 123-157. [↑](#footnote-ref-1)
2. Note that an approval poll will allow us to characterize approval for all definitional alternatives. Thus, if a definition proposed as an alternative to the one provided in Powell et al. (2012) is definition retained, we can characterize the approval level of the alternative relative to the definition already published. [↑](#footnote-ref-2)
3. In the event of a tie, the definition from Powell et al. (2012) will be carried forward. The record of the tie with the alternate definition will be included in the ancillary material for the strategy to retain this data. [↑](#footnote-ref-3)
