# Supplementary material for: A refined compilation of implementation strategies: results from the Expert Recommendations for Implementing Change (ERIC) project
Source: Implement Sci. 2015 Feb 12;10:21. doi: 10.1186/s13012-015-0209-1 (PMC4328074; doi:10.1186/s13012-015-0209-1)
Supplement: Additional file 4: — Expert Recommendations for Implementing Change (ERIC)—ballot for Round 3 of the modified Delphi process. This ballot specifies each of the strategies that were voted on in Round 3 of the modified Delphi. [file 13012_2015_209_MOESM4_ESM.docx]

**Additional File 4:**

Expert Recommendations for Implementing Change (ERIC) - Ballot for Round 3 of the Modified Delphi Process

It is highly recommended you prepare for the webinar using this form to help the pace of the vote quick. This will ensure quick documentation of consensus for terms where there is broad consensus, and leave time for discussion for terms lacking consensus. See the ERIC Voting Guide for a full description of the voting process.

|  | ***Strategies with one alternative proposed:*** | APPROVAL | | | | | RUN-OFF | | | | |
| --- | --- | --- | --- | --- | --- | --- | --- | --- | --- | --- | --- |
| 1. | **Tailor strategies**  ORIGINAL: Tailor the implementation effort to address barriers and to honor/accommodate stakeholder preferences that were identified through earlier data collection.  ALT 1: Tailor the implementation strategies to address barriers and leverage facilitators that were identified through earlier data collection. | O | ALT1 |  |  |  | O | ALT 1 |  |  |  |
| 2. | **Identify and prepare champions**  ORIGINAL: Cultivate relationships with people who will champion the clinical innovation and spread the word of the need for it.  ALT 1: Identify and prepare individuals who dedicate themselves to supporting, marketing, and driving through an implementation, overcoming indifference or resistance that the intervention may provoke in an organization. (Damschroeder et al., 2009) | O | ALT1 |  |  |  | O | ALT 1 |  |  |  |
| 3. | **Involve patients/consumers and family members**  ORIGINAL: Engage or include patients/consumers and families in all phases of the implementation effort, including training in the clinical innovation, and advocacy related to the innovation effort.  ALT 1: engage or include patients/consumers and families in the implementation effort | O | ALT1 |  |  |  | O | ALT 1 |  |  |  |
| 4. | **Provide ongoing consultation**  ORIGINAL: Provide clinicians with continued consultation with an expert in the clinical innovation.  ALT 1: Provide ongoing consultation with one or more experts in the strategies used to support implementing the innovation. | O | ALT1 |  |  |  | O | ALT 1 |  |  |  |
| 5. | **Use Data Experts**  ORIGINAL: Involve, hire and/or consult experts in data management to shape use of the considerable data that implementation efforts can generate.  ALT 1: Involve, hire, and/or consult experts to inform management and use of data generated by implementation efforts. | O | ALT1 |  |  |  | O | ALT 1 |  |  |  |

|  | ***Strategies with one alternative proposed****: (page 2)* | APPROVAL | | | | | RUN-OFF | | | | |
| --- | --- | --- | --- | --- | --- | --- | --- | --- | --- | --- | --- |
| 6. | **Use capitated payments**  ORIGINAL: Pay providers a set amount per patient/consumer for delivering clinical care.  ALT 1: pay providers or care systems a set amount per patient/consumer for delivering clinical care | O | ALT1 |  |  |  | O | ALT 1 |  |  |  |
| 7. | **Organize clinician implementation team meetings**  ORIGINAL: Develop and support teams of clinicians who are implementing the innovation and give them protected time to reflect on the implementation effort, share lessons learned, and support one another’s learning.  ALT 1: Provide clinicians with resources and coverage to participate in implementation team activities. | O | ALT1 |  |  |  | O | ALT 1 |  |  |  |
| 8. | **Intervene with patients/consumers to enhance uptake and adherence**  ORIGINAL: Intervene with patients/consumers to increase uptake of and adherence to clinical treatments  ALT 1: Develop strategies with patients to encourage and problem solve around adherence | O | ALT1 |  |  |  | O | ALT 1 |  |  |  |
| 9. | **Develop an implementation glossary**  ORIGINAL: Develop a glossary to promote common understanding about implementation among the different stakeholders.  ALT 1: Develop and distribute a list of terms describing the innovation, implementation, and the stakeholders in the organizational change. | O | ALT1 |  |  |  | O | ALT 1 |  |  |  |
| 10. | **Mandate change**  ORIGINAL: Declare that the innovation will be implemented.  ALT 1: Have leadership declare the priority of the innovation and determination to have it implemented | O | ALT1 |  |  |  | O | ALT 1 |  |  |  |
| 11. | **Assess for readiness and identify barriers and facilitators**  ORIGINAL: Assess various aspects of an organization to determine its degree of readiness to implement, barriers that may impede implementation, and strengths that can be used in the implementation effort.  ALT 1: Assess organizational readiness to adopt the innovation. Specify barriers and facilitators that inform your readiness assessment. | O | ALT1 |  |  |  | O | ALT 1 |  |  |  |

|  | ***Strategies with one alternative proposed****: (page 3)* | APPROVAL | | | | | RUN-OFF | | | | |
| --- | --- | --- | --- | --- | --- | --- | --- | --- | --- | --- | --- |
| 12. | **Start a dissemination organization**  ORIGINAL: Identify or start a separate organization that is responsible for disseminating the clinical innovation. It could be a for-profit or non-profit organization.  ALT 1: Create an organization that is both independent of the clinical organization and any entity that might financially or personally benefit from the innovation to disseminate the clinical innovation. This organization could be either for-profit or non-profit. | O | ALT1 |  |  |  | O | ALT 1 |  |  |  |
| 13. | **Develop disincentives**  ORIGINAL: Penalize providers financially for failure to implement or use the clinical innovation.  ALT 1: Provide financial disincentives for failure to implement or use the clinical innovations. | O | ALT1 |  |  |  | O | ALT 1 |  |  |  |

|  | ***Strategies with more than one alternative proposed:*** | APPROVAL | | | | | RUN-OFF | | | | |
| --- | --- | --- | --- | --- | --- | --- | --- | --- | --- | --- | --- |
| 14. | **Develop a formal implementation blueprint**  ORIGINAL: Develop a formal implementation blueprint that integrates multiple strategies from multiple levels or domains (e.g., staffing, funding, monitoring) using multiple theories or the use of an explicit theoretical framework. Use and update this plan to guide the implementation effort over time.  ALT 1: Develop a formal implementation blueprint. Use and update this plan to guide the implementation effort over time.   ALT 2: Develop a formal implementation blueprint that integrates multiple strategies from multiple levels or domains (e.g., staffing, funding, monitoring) using relevant theory and the results of pre-implementation barrier/facilitator assessments. Use and update this plan to guide the implementation effort over time.  ALT 3: Develop a formal implementation blueprint that includes all goals and strategies. The blueprint should include: 1) aim/purpose of the implementation; 2) scope of the change (e.g., what organizational units are affected); 3) timeframe and milestones; and 4) appropriate performance/progress measures. Use and update this plan to guide the implementation effort over time. | O | ALT1 | ALT2 | ALT3 |  | O | ALT 1 | ALT 2 | ALT 3 |  |
| 15. | **Create a learning collaborative**  ORIGINAL: Develop and use groups of providers or provider organizations that will implement the clinical innovation and develop ways to learn from one another to foster better implementation.  ALT 1: Facilitate the formation of groups of providers or provider organizations and foster a collaborative learning environment to improve implementation of the clinical innovation.  ALT 2: Develop and support ways for people involved in the implementation to learn from one another to promote continuing improvements in implementation effectiveness. | O | ALT1 | ALT2 |  |  | O | ALT 1 | ALT 2 |  |  |

|  | ***Strategies with more than one alternative proposed:*** *(page 2)* | APPROVAL | | | | | RUN-OFF | | | | |
| --- | --- | --- | --- | --- | --- | --- | --- | --- | --- | --- | --- |
| 16. | **Shadow other experts**  ORIGINAL: Have clinicians shadow other clinicians who are experts or knowledgeable in the clinical innovation and have implemented it.  ALT 1: Have clinicians directly observe other clinicians who are experts or knowledgeable in the clinical innovation.  ALT 2: Have individuals directly observe others who are experts or knowledgeable in the practice change/innovation and who have ideally implemented it.  ALT 3: Provide ways for key individuals to directly observe experienced people engage with or use the targeted practice change/innovation. | O | ALT1 | ALT2 | ALT3 |  | O | ALT 1 | ALT 2 | ALT 3 |  |
| 17. | **Change physical structure and equipment**  ORIGINAL: Change the physical structure and equipment (changing the layout of a room, adding equipment).  ALT 1: Change the physical structure or equipment (e.g., changing the layout of a room, adding equipment) in ways that support the innovation.  ALT 2: Involve the users of structure and equipment to contribute to the redesign of the workflow and/or the features of the new equipment.  ALT 3: Evaluate current configurations and adapt, as needed, the physical structure and/or equipment (e.g., changing the layout of a room, adding equipment) to best accommodate the targeted innovation. | O | ALT1 | ALT2 | ALT3 |  | O | ALT 1 | ALT 2 | ALT 3 |  |
| 18. | **Facilitate relay of clinical data to providers**  ORIGINAL: Collect new clinical information from the patient/consumer and relay it to the provider outside the traditional clinical encounter to prompt the provider to use the clinical innovation.  ALT 1: Collect clinical information from the patient/consumer and/or their medical records and send to the provider outside the traditional clinical encounter to prompt the provider to use the clinical innovation.  ALT 2: Provide as close to real-time data as possible about key measures of process/outcomes using integrated modes/channels of communication in a way that promotes use of the targeted innovation. | O | ALT1 | ALT2 |  |  | O | ALT 1 | ALT 2 |  |  |

|  | ***Strategies with more than one alternative proposed:*** *(page 3)* | APPROVAL | | | | | RUN-OFF | | | | |
| --- | --- | --- | --- | --- | --- | --- | --- | --- | --- | --- | --- |
| 19. | **Use advisory boards & work groups**  ORIGINAL: Involve multiple kinds of stakeholders in a group to oversee implementation efforts and make recommendations.  ALT 1: Involve multiple kinds of stakeholders in a group to provide input and advice on implementation efforts and make recommendations.  ALT 2: Involve multiple kinds of stakeholders in a group to inform implementation efforts by providing input, advice, recommendations and/or oversight.  ALT 3: Involve multiple kinds of stakeholders in a group to inform implementation efforts.  ALT 4: Create and engage a formal group of multiple kinds of stakeholders to provide input and advice on implementation efforts and to elicit recommendations for improvements. | O | ALT1 | ALT2 | ALT3 | ALT 4 | O | ALT 1 | ALT 2 | ALT 3 | ALT4 |
| 20. | **Purposefully reexamine the implementation**  ORIGINAL: Obtain commitment from stakeholders to use monitoring to adjust practice and strategies to continuously improve the implementation effort and delivery of the clinical innovation.  ALT 1: Monitor progress and adjust implementation as needed  ALT 2: Use appropriate measures to monitor progress of implementation and make refinements to the Blueprint and changes in execution, as needed, to improve or refine implementation strategies.  ALT 3: Monitor progress and adjust clinical practices and implementation strategies to continuously improve the quality of care. | O | ALT1 | ALT2 | ALT3 |  | O | ALT 1 | ALT 2 | ALT 3 |  |
| 21. | **Model and simulate change**  ORIGINAL: Model or simulate the change that will be implemented prior to implementation.  ALT 1: Model or simulate the innovation (or components of the innovation) prior to implementation of the intervention.  ALT 2: Model or simulate the innovation that will be implemented. | O | ALT1 | ALT2 |  |  | O | ALT 1 | ALT 2 |  |  |

|  | ***Strategies proposed during Delphi survey*** | APPROVAL | | | | | RUN-OFF | | | | |
| --- | --- | --- | --- | --- | --- | --- | --- | --- | --- | --- | --- |
| 22. | **Promote Network Weaving**  DEFINITION: Identify and build on existing high quality working relationships and networks within and outside the organization, organizational units, teams, etc. to promote information sharing, collaborative problem-solving, and building a shared vision/goal related to implementing the targeted innovation. | Voting only to accept or reject the proposed strategy (no alternatives) | | | | | DEF | REJECT |  |  |  |
| 23. | **Provide local technical assistance**  DEFINITION: Develop and use a system to deliver technical assistance focused on implementation issues using local personnel. | Voting only to accept or reject the proposed strategy (no alternatives) | | | | | DEF | REJECT |  |  |  |
| 24. | **Promote adaptability**  DEFINITION: Identify the ways a clinical innovation can be tailored to meet local needs and clarify which elements of the innovation must be maintained to preserve fidelity.  ALT 1: Identify the ways in which a clinical innovation can be tailored to meet local needs and highlight the elements that are central to fidelity to the innovation. | DEF | ALT 1 | REJECT |  |  | DEF | ALT 1 |  |  |  |
| 25. | **Identify early adopters**  DEFINITION: Identify early adopters at the local site to review their experiences with the practice innovation.  ALT 1: Identify early adopters at the local site to learn from their experiences with the practice innovation. | DEF | ALT 1 | REJECT |  |  | DEF | ALT 1 |  |  |  |
| 26. | **Facilitation**  DEFINITION: A process of interactive problem-solving and support which occurs in a context of a recognized need for improvement and a supportive interpersonal relationship.  (Stetler et al. Implementation Science 2006; 1:23)  ALT 1: The process of enabling (or making easier) the implementation of evidence into practice. (Harvey et al., 2002)  ALT 2: An expert who models, teaches, or leads interactive problem-solving and support within a context of a recognized need for improvement and a supportive interpersonal relationship. | DEF | ALT 1 | ALT 2 | REJECT |  | DEF | ALT 1 | ALT 2 |  |  |
