# Supplementary material for: A refined compilation of implementation strategies: results from the Expert Recommendations for Implementing Change (ERIC) project
Source: Implement Sci. 2015 Feb 12;10:21. doi: 10.1186/s13012-015-0209-1 (PMC4328074; doi:10.1186/s13012-015-0209-1)
Supplement: Additional file 5: — Expert Recommendations for Implementing Change (ERIC)—results from modified Delphi Round 3 voting. This document lists the voting results from modified Delphi Round 3. [file 13012_2015_209_MOESM5_ESM.docx]

**Additional File 5:**

Expert Recommendations for Implementing Change (ERIC) –

Results from Modified Delphi Round 3 Voting

|  |  | Voting Percentages | |
| --- | --- | --- | --- |
| Terms | Definitions | Approval | Runoff |
| *Access new funding* | Access new or existing money to facilitate the implementation. | nc | -- |
| *Alter incentive/allowance structures* | Work to incentivize the adoption and implementation of the clinical innovation. | nc | -- |
| *Alter patient/consumer fees* | Create fee structures where patients/consumers pay less for preferred treatments (the clinical innovation) and more for less-preferred treatments. | nc | -- |
| *Assess for readiness and identify barriers & facilitators* | ORIGINAL: Assess various aspects of an organization to determine its degree of readiness to implement, barriers that may impede implementation, and strengths that can be used in the implementation effort. | 69* | -- |
|  | ALT 1: Assess organizational readiness to adopt the innovation. Specify barriers and facilitators that inform your readiness assessment. | 51 | -- |
| *Audit and provide feedback* | Collect and summarize clinical performance data over a specified time period and give it to clinicians and administrators to monitor, evaluate, and modify provider behavior. | nc | -- |
| *Build a coalition* | Recruit and cultivate relationships with partners in the implementation effort. | nc | -- |
| *Capture and share local knowledge* | Capture local knowledge from implementation sites on how implementers and clinicians made something work in their setting and then share it with other sites. | nc | -- |
| *Centralize technical assistance* | Develop and use a centralized system to deliver technical assistance focused on implementation issues. | nc | -- |
| *Change accreditation or membership requirements* | Strive to alter accreditation standards so that they require or encourage use of the clinical innovation. . Work to alter membership organization requirements so that those who want to affiliate with the organization are encouraged or required to use the clinical innovation. | nc | -- |
| *Change liability laws* | Participate in liability reform efforts that make clinicians more willing to deliver the clinical innovation. | nc | -- |
| *Change physical structure and equipment* | ORIGINAL: Change the physical structure and equipment (changing the layout of a room, adding equipment). | 3 | -- |
|  | ALT 1: Change the physical structure or equipment (e.g., changing the layout of a room, adding equipment) in ways that support the innovation. | 37 | -- |
|  | ALT 2: Involve the users of structure and equipment to contribute to the redesign of the workflow and/or the features of the new equipment. | 34 | -- |
|  | ALT 3: Evaluate current configurations and adapt, as needed, the physical structure and/or equipment (e.g., changing the layout of a room, adding equipment) to best accommodate the targeted innovation. | 80* | -- |
| *Change record systems* | Change records systems to allow better assessment of implementation or of outcomes of the implementation. | nc | -- |
| *Change service sites* | Change the location of clinical service sites to increase access. | nc | -- |
| *Conduct cyclical small tests of change* | Implement changes in a cyclical fashion using small tests of change before taking changes system-wide. Tests of change benefit from systematic measurement, and results of the tests of change are studied for insights on how to do better. This process continues serially over time, and refinement is added with each cycle. | nc | -- |
| *Conduct educational meetings* | Hold meetings targeted toward different stakeholder groups (e.g., providers, administrators, other organizational stakeholders, and community, patient/consumer, and family stakeholders) to teach them about the clinical innovation. | nc | -- |
| *Conduct educational outreach visits* | Have a trained person meet with providers in their practice settings to educate providers about the clinical innovation with the intent of changing the provider’s practice. | nc | -- |
| *Conduct local consensus discussions* | Include local providers and other stakeholders in discussions that address whether the chosen problem is important and whether the clinical innovation to address it is appropriate. | nc | -- |
| *Conduct local needs assessment* | Collect and analyze data related to the need for the innovation. | nc | -- |
| *Conduct ongoing training* | Plan for and conduct training in the clinical innovation in an ongoing way. | nc | -- |
| *Create a learning collaborative* | ORIGINAL: Develop and use groups of providers or provider organizations that will implement the clinical innovation and develop ways to learn from one another to foster better implementation. | 42 | -- |
|  | ALT 1: Facilitate the formation of groups of providers or provider organizations and foster a collaborative learning environment to improve implementation of the clinical innovation. | 64* | -- |
|  | ALT 2: Develop and support ways for people involved in the implementation to learn from one another to promote continuing improvements in implementation effectiveness. | 53 | -- |
| *Create new clinical teams* | Change who serves on the clinical team, adding different disciplines and different skills to make it more likely that the clinical innovation is delivered or more successful. | nc | -- |
| *Create or change credentialing and/or licensure standards* | Create an organization that certifies clinicians in the innovation or encourages an existing organization to do so. Change governmental professional certification or licensure requirements to include delivering the innovation. Work to alter continuing education requirements to shape professional practice toward the innovation. | nc | -- |
| *Develop a formal implementation blueprint* | ORIGINAL: Develop a formal implementation blueprint that integrates multiple strategies from multiple levels or domains (e.g., staffing, funding, monitoring) using multiple theories or the use of an explicit theoretical framework. Use and update this plan to guide the implementation effort over time. | 12 | -- |
|  | ALT 1: Develop a formal implementation blueprint. Use and update this plan to guide the implementation effort over time. | 26 | -- |
|  | ALT 2: Develop a formal implementation blueprint that integrates multiple strategies from multiple levels or domains (e.g., staffing, funding, monitoring) using relevant theory and the results of pre-implementation barrier/facilitator assessments. Use and update this plan to guide the implementation effort over time. | 53 | -- |
|  | ALT 3: Develop a formal implementation blueprint that includes all goals and strategies. The blueprint should include: 1) aim/purpose of the implementation; 2) scope of the change (e.g., what organizational units are affected); 3) timeframe and milestones; and 4) appropriate performance/progress measures. Use and update this plan to guide the implementation effort over time. | 71* | -- |
| *Develop academic partnerships* | Partner with a university or academic unit for the purposes of shared training and bringing research skills to an implementation project. | nc | -- |
| *Develop an implementation glossary* | ORIGINAL: Develop a glossary to promote common understanding about implementation among the different stakeholders. | 46 | -- |
|  | ALT 1: Develop and distribute a list of terms describing the innovation, implementation, and the stakeholders in the organizational change. | 69* | -- |
| *Develop and implement tools for quality monitoring* | Develop, test, and introduce into quality-monitoring systems the right input—the appropriate language, protocols, algorithms, standards, and measures (of processes, patient/consumer outcomes, and implementation outcomes) that are often specific to the innovation being implemented. | nc | -- |
| *Develop and organize quality monitoring systems* | Develop and organize systems and procedures that monitor clinical processes and/or outcomes for the purpose of quality assurance and improvement. | nc | -- |
| *Develop disincentives* | ORIGINAL: Penalize providers financially for failure to implement or use the clinical innovation. | 29 | -- |
|  | ALT 1: Provide financial disincentives for failure to implement or use the clinical innovations. | 82* | -- |
| *Develop educational materials* | Develop and format manuals, toolkits and other supporting materials in ways that make it easier for stakeholders to learn about the innovation and for clinicians to learn how to deliver the clinical innovation. | nc | -- |
| *Develop resource sharing agreements* | Develop partnerships with organizations that have resources needed to implement the innovation. | nc | -- |
| *Distribute educational materials* | Distribute educational materials (including guidelines, manuals and toolkits) in person, by mail, and/or electronically. | nc | -- |
| *Facilitate relay of clinical data to providers* | ORIGINAL: Collect new clinical information from the patient/consumer and relay it to the provider outside the traditional clinical encounter to prompt the provider to use the clinical innovation. | 9 | -- |
|  | ALT 1: Collect clinical information from the patient/consumer and/or their medical records and send to the provider outside the traditional clinical encounter to prompt the provider to use the clinical innovation. | 51 | -- |
|  | ALT 2: Provide as close to real-time data as possible about key measures of process/outcomes using integrated modes/channels of communication in a way that promotes use of the targeted innovation. | 63* | -- |
| *Facilitation* | NEW: A process of interactive problem-solving and support which occurs in a context of a recognized need for improvement and a supportive interpersonal relationship. | 44 | 67* |
|  | ALT 1: The process of enabling (or making easier) the implementation of evidence into practice. | 38 | 20 |
|  | ALT 2: An expert who models, teaches, or leads interactive problem-solving and support within a context of a recognized need for improvement and a supportive interpersonal relationship. | 34 | 13 |
|  | REJECT | 16 | -- |
| *Fund and contract for the clinical innovation* | Governments and other payers of services issue requests for proposals to deliver the innovation, use contracting processes to motivate providers to deliver the clinical innovation, and develop new funding formulas that make it more likely that providers will deliver the innovation. | nc | -- |
| *Identify and prepare champions* | ORIGINAL: Cultivate relationships with people who will champion the clinical innovation and spread the word of the need for it. | 35 | -- |
|  | ALT 1: Identify and prepare individuals who dedicate themselves to supporting, marketing, and driving through an implementation, overcoming indifference or resistance that the intervention may provoke in an organization. | 82* | -- |
| *Identify early adopters* | NEW: Identify early adopters at the local site to review their experiences with the practice innovation. | 18 | -- |
|  | ALT 1: Identify early adopters at the local site to learn from their experiences with the practice innovation. | 88* | -- |
|  | REJECT | 6 | -- |
| *Increase demand* | Attempt to influence the market for the clinical innovation to increase competition intensity and to increase the maturity of the market for the clinical innovation. | nc | -- |
| *Inform local opinion leaders* | Inform providers identified by colleagues as opinion leaders or ‘educationally influential’ about the clinical innovation in the hopes that they will influence colleagues to adopt it. | nc | -- |
| *Intervene with patients/consumers to enhance uptake and adherence* | ORIGINAL: Intervene with patients/consumers to increase uptake of and adherence to clinical treatments | 51 | -- |
|  | ALT 1: Develop strategies with patients to encourage and problem solve around adherence. | 77* | -- |
| *Involve executive boards* | Involve existing governing structures (e.g., boards of directors, medical staff boards of governance) in the implementation effort, including the review of data on implementation processes. | nc | -- |
| *Involve patients/consumers and family members* | ORIGINAL: Engage or include patients/consumers and families in all phases of the implementation effort, including training in the clinical innovation, and advocacy related to the innovation effort. | 47 | -- |
|  | ALT 1: Engage or include patients/consumers and families in the implementation effort. | 61* | -- |
| *Make billing easier* | Make it easier to bill for the clinical innovation. | nc | -- |
| *Make training dynamic* | Vary the information delivery methods to cater to different learning styles work contexts, and shape the training in the innovation to be interactive. | nc | -- |
| *Mandate change* | ORIGINAL: Declare that the innovation will be implemented. | 17 | -- |
|  | ALT 1: Have leadership declare the priority of the innovation and determination to have it implemented | 91* | -- |
| *Model and simulate change* | ORIGINAL: Model or simulate the change that will be implemented prior to implementation. | 49 | 82* |
|  | ALT 1: Model or simulate the innovation (or components of the innovation) prior to implementation of the intervention. | 54 | 15 |
|  | ALT 2: Model or simulate the innovation that will be implemented. | 31 | 3 |
| *Obtain and use patients/consumers and family feedback* | Develop strategies to increase patient/consumer and family feedback on the implementation effort. | nc | -- |
| *Obtain formal commitments* | Obtain written commitments from key partners that state what they will do to implement the innovation. | nc | -- |
| *Organize clinician implementation team meetings* | ORIGINAL: Develop and support teams of clinicians who are implementing the innovation and give them protected time to reflect on the implementation effort, share lessons learned, and support one another’s learning. | 75* | -- |
|  | ALT 1: Provide clinicians with resources and coverage to participate in implementation team activities. | 44 | -- |
| *Place innovation on fee for service lists/formularies* | Work to place the clinical innovation on lists of actions for which providers can be reimbursed (e.g., a drug is placed on a formulary, a procedure is now reimbursable). | nc | -- |
| *Prepare patients/consumers to be active participants* | Prepare patients/consumers to be active in their care, to ask questions, and specifically to inquire about care guidelines, the evidence behind clinical decisions, or about available evidence-supported treatments. | nc | -- |
| *Promote adaptability* | NEW: Identify the ways a clinical innovation can be tailored to meet local needs and clarify which elements of the innovation must be maintained to preserve fidelity. | 66 | 91* |
|  | ALT 1: Identify the ways in which a clinical innovation can be tailored to meet local needs and highlight the elements that are central to fidelity to the innovation. | 66 | 9 |
|  | REJECT | 0 | -- |
| *Promote network weaving* | NEW: Identify and build on existing high quality working relationships and networks within and outside the organization, organizational units, teams, etc. to promote information sharing, collaborative problem-solving, and building a shared vision/goal related to implementing the targeted innovation. | 71* | -- |
|  | REJECT | 29 | -- |
| *Provide clinical supervision* | Provide clinicians with ongoing supervision focusing on the innovation. Provide training for clinical supervisors who will supervise clinicians who provide the innovation. | nc | -- |
| *Provide local technical assistance* | NEW: Develop and use a system to deliver technical assistance focused on implementation issues using local personnel. | 73* | -- |
|  | REJECT | 27 | -- |
| *Provide ongoing consultation* | ORIGINAL: Provide clinicians with continued consultation with an expert in the clinical innovation. | 42 | -- |
|  | ALT 1: Provide ongoing consultation with one or more experts in the strategies used to support implementing the innovation. | 78* | -- |
| *Purposefully reexamine the implementation* | ORIGINAL: Obtain commitment from stakeholders to use monitoring to adjust practice and strategies to continuously improve the implementation effort and delivery of the clinical innovation. | 23 | -- |
|  | ALT 1: Monitor progress and adjust implementation as needed | 31 | -- |
|  | ALT 2: Use appropriate measures to monitor progress of implementation and make refinements to the Blueprint and changes in execution, as needed, to improve or refine implementation strategies. | 49 | -- |
|  | ALT 3: Monitor progress and adjust clinical practices and implementation strategies to continuously improve the quality of care. | 60* | -- |
| *Recruit, designate, and train for leadership* | Recruit, designate, and train leaders for the change effort. | nc | -- |
| *Remind clinicians* | Develop reminder systems designed to help clinicians to recall information and/or prompt them to use the clinical innovation. | nc | -- |
| *Revise professional roles* | Shift and revise roles among professionals who provide care and redesign job characteristics. | nc | -- |
| *Shadow other experts* | ORIGINAL: Have clinicians shadow other clinicians who are experts or knowledgeable in the clinical innovation and have implemented it. | 26 | -- |
|  | ALT 1: Have clinicians directly observe other clinicians who are experts or knowledgeable in the clinical innovation. | 41 | -- |
|  | ALT 2: Have individuals directly observe others who are experts or knowledgeable in the practice change/innovation and who have ideally implemented it. | 50 | -- |
|  | ALT 3: Provide ways for key individuals to directly observe experienced people engage with or use the targeted practice change/innovation. | 65* | -- |
| *Stage implementation scale up* | Phase implementation efforts by starting with small pilots or demonstration projects and gradually moving to a system wide rollout. | nc | -- |
| *Start a dissemination organization* | ORIGINAL: Identify or start a separate organization that is responsible for disseminating the clinical innovation. It could be a for-profit or non-profit organization. | 61* | -- |
|  | ALT 1: Create an organization that is both independent of the clinical organization and any entity that might financially or personally benefit from the innovation to disseminate the clinical innovation. This organization could be either for-profit or non-profit. | 56 | -- |
| *Tailor strategies* | ORIGINAL: Tailor the implementation effort to address barriers and to honor/accommodate stakeholder preferences that were identified through earlier data collection. | 44 | -- |
|  | ALT 1: Tailor the implementation strategies to address barriers and leverage facilitators that were identified through earlier data collection. | 71* | -- |
| *Use advisory boards and workgroups* | ORIGINAL: Involve multiple kinds of stakeholders in a group to oversee implementation efforts and make recommendations. | 22 | -- |
|  | ALT 1: Involve multiple kinds of stakeholders in a group to provide input and advice on implementation efforts and make recommendations. | 42 | -- |
|  | ALT 2: Involve multiple kinds of stakeholders in a group to inform implementation efforts by providing input, advice, recommendations and/or oversight. | 47 | -- |
|  | ALT 3: Involve multiple kinds of stakeholders in a group to inform implementation efforts. | 17 | -- |
|  | ALT 4: Create and engage a formal group of multiple kinds of stakeholders to provide input and advice on implementation efforts and to elicit recommendations for improvements. | 67* | -- |
| *Use an implementation advisor* | Seek guidance from experts in implementation. | nc | -- |
| *Use capitated payments* | ORIGINAL: Pay providers a set amount per patient/consumer for delivering clinical care. | 17 | -- |
|  | ALT 1: Pay providers or care systems a set amount per patient/consumer for delivering clinical care. | 97* | -- |
| *Use data experts* | ORIGINAL: Involve, hire and/or consult experts in data management to shape use of the considerable data that implementation efforts can generate. | 39 | -- |
|  | ALT 1: Involve, hire, and/or consult experts to inform management on the use of data generated by implementation efforts. | 92* | -- |
| *Use data warehousing techniques* | Integrate clinical records across facilities and organizations to facilitate implementation across systems. | nc | -- |
| *Use mass media* | Use media to reach large numbers of people to spread the word about the clinical innovation. | nc | -- |
| *Use other payment schemes* | Introduce payment approaches (in a catch-all category). | nc | -- |
| *Use train-the-trainer strategies* | Train designated clinicians or organizations to train others in the clinical innovation. | nc | -- |
| *Visit other sites* | Visit sites where a similar implementation effort has been considered successful. | nc | -- |
| *Work with educational institutions* | Encourage educational institutions to train clinicians in the innovation. | nc | -- |

*Notes*: Strategies for which no alternative definitions were introduced are labeled as not contested (nc) in the approval vote column. The definition with the winning number of votes is marked with an asterisk (*). Runoff voting was only held if a clear supermajority winner (≥60%) was not obtained in the approval vote. For each strategy and round of voting there were a small number of abstainers, with the percentage of participants casting votes ranging from 83% to 94%.
