# Supplementary material for: A refined compilation of implementation strategies: results from the Expert Recommendations for Implementing Change (ERIC) project
Source: Implement Sci. 2015 Feb 12;10:21. doi: 10.1186/s13012-015-0209-1 (PMC4328074; doi:10.1186/s13012-015-0209-1)
Supplement: Additional file 6: — Expert Recommendations for Implementing Change (ERIC)—discrete implementation strategy compilation with ancillary material. This file contains the final ERIC discrete strategy compilation and the associated ancillary material. [file 13012_2015_209_MOESM6_ESM.docx]

**Additional File 6:**

Expert Recommendations for Implementing Change (ERIC) –

Discrete Implementation Strategy Compilation with Ancillary Material

| **Access new funding**  Access new or existing money to facilitate the implementation.  ***Ancillary Material:***  Accessing new funding sources could involve new uses of existing money, accessing block grants, shifting funding from one program to another, cost-sharing, passing new taxes, raising private funds, or applying for grants. These monies may be used to fund the delivery of a clinical innovation, or to support other time limited actions needed for initial implementation, such as to purchase material or logistical support, training, and consultations. |
| --- |
| **Alter incentive/allowance structures**  Work to incentivize the adoption and implementation of the clinical innovation.  ***Ancillary Material:***  Incentives may be based on the performance of individual clinicians or larger performance units at the organizational level. The incentive could be in the form of an increased rate of pay to cover the incremental costs associated with implementing the clinical innovation. The incentive could be through loan reduction or forgiveness to clinicians to learn an innovation. This category of financial strategies also includes the elimination of any perverse incentives that become a barrier to receiving appropriate care. An incentive suggests the payment is tied to performing a clinical action or improving outcomes. An allowance suggests that the clinician or organization is not required to perform the clinical action or meet the performance standard. |
| **Alter patient/consumer fees**  Create fee structures where patients/consumers pay less for preferred treatments (the clinical innovation) and more for less-preferred treatments.  ***Ancillary Material:***  None |
| **Assess for readiness and identify barriers and facilitators**  Assess various aspects of an organization to determine its degree of readiness to implement, barriers that may impede implementation, and strengths that can be used in the implementation effort.  ***Ancillary Material:***  Readiness assessments may focus on agency finances, staffing levels, and other material or logistical resources needed, or available, to support the implementation effort. Further this assessment may also focus on leadership support, the organizational priority for change, and the presence of successful experience with quality improvement techniques and change management. Additional aspects for assessment may include other services provided, as well as community support, stakeholder attitudes, and beliefs and perceptions of evidence for the innovation or change. Rationale for current practices, organizational climate and culture, structure, decision-making styles, and the perceived needs of frontline stakeholders to implement the change or innovation (consider adaptation needs and limits) are also important aspects to consider in this assessment. Readiness assessments can be used to vet, eliminate, or prioritize implementation sites. More so, the assessment can help make internal decisions about whether to go ahead with an implementation initiative. Some barriers can be difficult to observe prior to implementation. Specific measures have been created to assess readiness for change, which may be useful (e.g., [1–3]). |
| **Audit and provide feedback**  Collect and summarize clinical performance data over a specified time period and give it to clinicians and administrators to monitor, evaluate, and modify provider behavior.  ***Ancillary Material:***  The information may be obtained from a variety of sources, including medical records, computerized databases, observation, or feedback from patients. Performance evaluations may also be considered as audit and feedback data if the evaluation included specific information on clinical performance. Feedback summaries may include recommendations. Feedback may be displayed publicly, and often involves comparisons to peers or to local, state, national, or international norms. Feedback may be designed to guide a clinician in improving fidelity. It should also be noted that audit and feedback data can be helpful in promoting the continuation of intended behavior. Performance data may include process variables, outcomes, or fidelity measures. Feedback can include mandatory performance measures, which are related to benchmarks from the literature or normative data within an organization or industry. |
| **Build a coalition**  Recruit and cultivate relationships with partners in the implementation effort.  ***Ancillary Material:***  Partnerships can develop around cost-sharing, shared resources, shared training, and the division of responsibilities among partners. This work may proceed naturally from local consensus discussions. Coalition members commonly have defined roles in the implementation effort. |
| **Capture and share local knowledge**  Capture local knowledge from implementation sites on how implementers and clinicians made something work in their setting and then share it with other sites.  ***Ancillary Material:***  This strategy is often coordinated with centralized technical assistance and learning collaboratives. There are multiple techniques for capturing local knowledge, which could be presented in multiple formats. For example, short YouTube videos could be created that document testimonials from clinicians who have successfully used a given innovation. Another example would be maintaining a running list of a team's response to specific implementation barriers that could be shared readily through a platform like GoogleDocs or Microsoft SharePoint. Additional techniques can be found at [www.liberatingstructures.com](http://www.liberatingstructures.com). |
| **Centralize technical assistance**  Develop and use a centralized system to deliver technical assistance focused on implementation issues.  ***Ancillary Material:***  This could be the designation of a lead technical assistance organization (could also be responsible for training). The lead technical assistance entity can develop other mechanisms (e.g., call-in lines or websites) in order to share information on how to best implement the clinical innovation. |
| **Change accreditation or membership requirements**  Strive to alter accreditation standards so that they require or encourage use of the clinical innovation. Work to alter membership organization requirements so that those who want to affiliate with the organization are encouraged or required to use the clinical innovation.  ***Ancillary Material:***  None |
| **Change liability laws**  Participate in liability reform efforts that make clinicians more willing to deliver the clinical innovation.  ***Ancillary Material:***  Liability reform can also make clinicians less willing to deliver alternatives to the clinical innovation. |
| **Change physical structure and equipment**  Evaluate current configurations and adapt, as needed, the physical structure and/or equipment (e.g., changing the layout of a room, adding equipment) to best accommodate the targeted innovation.  ***Ancillary Material:***  None |
| **Change record systems**  Change records systems to allow better assessment of implementation or clinical outcomes.  ***Ancillary Material:***  These changes may include modifying the format of progress notes and treatment plans to reflect the innovation (evidence-based practice) being implemented. |
| **Change service sites**  Change the location of clinical service sites to increase access.  ***Ancillary Material:***  Changing service sites can include collocating different services to better implement complex clinical innovations that require multiple disciplines or services, telemedicine, or bringing the services to the client in their home, the community, or other clinically relevant settings, such as busy public spaces for a client with PTSD. |
| **Conduct cyclical small tests of change**  Implement changes in a cyclical fashion using small tests of change before taking changes system-wide. Tests of change benefit from systematic measurement, and results of the tests of change are studied for insights on how to do better. This process continues serially over time, and refinement is added with each cycle.  ***Ancillary Material:***  Two common small tests of change cycling strategies are “Plan-Do-Study-Act” from Deming’s quality management work [4], and six sigma’s Define- Measure- Analyze-Improve-Control sequence [5]. |
| **Conduct educational meetings**  Hold meetings targeted toward different stakeholder groups (e.g., providers, administrators, other organizational stakeholders, and community, patient/consumer, and family stakeholders) to teach them about the clinical innovation.  ***Ancillary Material:***  The content of the education may include information regarding what to expect as implementation moves forward. It is useful to ensure that meeting attendees are relatively homogeneous so that the education can be targeted toward the stakeholder group’s needs. For example, some educational meetings may inform the stakeholder group about the clinical innovation in a way intended to increase demand, while others may preview the clinical innovation for providers and administrators. It is often useful to have recordings or other materials from the educational meetings available to those who cannot attend the meetings (e.g., those covering patient care at the time of the meeting, new hires subsequent to the meeting). |
| **Conduct educational outreach visits**  Have a trained person meet with providers in their practice settings to educate providers about the clinical innovation with the intent of changing the provider’s practice.  ***Ancillary Material:***  Visits to the site may be in-person or virtually via the Internet. Some initiatives may require regular educational outreach as part of maintaining the innovation/practice change. Academic detailing is another commonly used term, although academic detailing typically involves many additional discrete implementation strategies (e.g., conduct ongoing training, modeling, developing and distributing educational materials; [21, 22]). |
| **Conduct local consensus discussions**  Include local providers and other stakeholders in discussions that address whether the chosen problem is important and whether the clinical innovation to address it is appropriate.  ***Ancillary Material:***  Identify stakeholders relevant to each project. Further, with each project, there will be a need to identify whether the goal of the consensus discussion is to characterize consensus or build consensus. Utilizing community based participatory research principles may be relevant to many innovations. Notably, the chosen problem needs to be a high enough priority, compared to other problems, that attention and resources will be dedicated to addressing the problem. |
| **Conduct local needs assessment**  Collect and analyze data related to the need for the innovation.  ***Ancillary Material:***  This assessment could be focused on:   - Outcomes of usual care - Process of care - Description of usual care and its distance from evidence-based care (e.g., gaps in care) - Opinions from stakeholders (including patients) on (a) barriers and facilitators to the desired outcome (e.g., recovery from mental illness), (b) the need for any innovation (i.e., tension for change), (c) the need for a specific innovation, or (d) the special considerations for delivering the innovation in the local context.   Common needs assessment methods include surveys, focus groups, key informant interviews, direct observation, and data mining of administrative records utilized to identify target populations, as well as identify baseline care process and outcome clinical care data. If the change involves multiple sites or facilities, then it is necessary to examine practice variation across facilities, and outline strategies for the needs assessment to support a standardized approach across sites. Collecting data from a random sample of stakeholders may be necessary to reduce response bias and decrease chances that the level of need is not over or under-estimated. |
| **Conduct ongoing training**  Plan for and conduct training in the clinical innovation in an ongoing way.  ***Ancillary Material:***  This can include follow-up training, advanced training, booster training, purposefully spaced training, training to competence, integration of off the- job and on-the-job training, structured supervision, the introduction of concepts in a specific sequence to ensure mastery, and trainings based on the level of clinician knowledge. Ongoing training efforts need to reach across shifts and accommodate staff turnover, as well as rotating staff (e.g., residents). Trainings can be in-person, on the web, or technology-assisted (e.g., simulation lab training), and may focus on individuals or involve groups. When planning for ongoing training, it is important to describe the training components, including the timing and frequency of trainings. Issues related to the dynamics of training can be found in the strategy, make training dynamic. |
| **Create a learning collaborative**  Facilitate the formation of groups of providers or provider organizations and foster a collaborative learning environment to improve implementation of the clinical innovation.  ***Ancillary Material:***  There are several approaches to this in the literature including peer consultation networks, online communities of practice, quality circles, and learning collaboratives. Groups may meet in person or interact using a wide variety of media. The inclusion of a quality manager within the collaborative may be useful. Positive deviance approaches use “discovery and action dialogue” among peers to promote collaborative learning [23–25].  Resources specific to learning collaboratives include:  The Health Resources and Services Administration (HRSA) [26]  The Institute for Healthcare Improvement (IHI) [27, 28]  Key terms for searching literature specific to collaborative learning include:  learning community, learning network, and community of practice. |
| **Create new clinical teams**  Change who serves on the clinical team, adding different disciplines and different skills to make it more likely that the clinical innovation is delivered (or is more successfully delivered).  ***Ancillary Material:***  None |
| **Create or change credentialing and/or licensure standards**  Create an organization that certifies clinicians in the innovation or encourage an existing organization to do so. Change governmental professional certification or licensure requirements to include delivering the innovation. Work to alter continuing education requirements to shape professional practice toward the innovation.  ***Ancillary Material:***  None |
| **Develop a formal implementation blueprint**  Develop a formal implementation blueprint that includes all goals and strategies. The blueprint should include: 1) aim/purpose of the implementation; 2) scope of the change (e.g., what organizational units are affected); 3) timeframe and milestones; and 4) appropriate performance/progress measures. Use and update this plan to guide the implementation effort over time.  ***Ancillary Material:***  The implementation blueprint or manual may be informed by one or more theories or conceptual frameworks and/or data from pre-implementation needs assessments. This blueprint can also provide a useful historical record of the implementation process, as well as provide a mechanism to track changes over time. The implementation blueprint is often useful to ensure feedback is received from prospective frontline users of the blueprint prior to implementation.  Consider coordinating this strategy with the development of a fidelity monitoring tool.  Issues to consider separately, especially for research purposes:   - Number and type of implementation strategies - Organizational levels involved—this can vary by type of intervention. It may be possible to do some interventions at the lowest level. Others may require top management. - Pre-implementation assessments would be separate step   Other examples of how to create an implementation blueprint include the CDC's Replicating Effective Programs [6, 7].  Examples of projects using a blueprint include:   - HI-TIDES [8] - Depression in a substance abusing population [9] - Mental health services in federally qualified health centers [10] |
| **Develop academic partnerships**  Partner with a university or academic unit for the purposes of shared training and bringing research skills to an implementation project.  ***Ancillary Material:***  HIPAA, and other legal limitations are common to encounter with academic partnerships. Formal relationships (e.g., contracts, MOUs) will be required in some instances. Not all academics have a full understanding of practice level stakeholder needs and this should be considered while developing this partnership. In settings where ‘research’ is not a commonly supported practice, evaluation or developmental evaluation may be more useful ways of characterize the activity [18]. |
| **Develop an implementation glossary**  Develop and distribute a list of terms describing the innovation, implementation, and the stakeholders in the organizational change.  ***Ancillary Material:***  When compiling a glossary, reflect as to whether the terms being introduced are essential. |
| **Develop and implement tools for quality monitoring**  Develop, test, and introduce into quality-monitoring systems the right input—the appropriate language, protocols, algorithms, standards, and measures (of processes, patient/consumer outcomes, and implementation outcomes) that are often specific to the innovation being implemented.  ***Ancillary Material:***  These tools should be flexible enough to reflect fidelity, even after adaptations to the setting or client. Performance sites can benefit when these tools are available locally, particularly to help clinicians develop a sense of ownership for the change process. Quality monitoring tools can be coordinated with other strategies to encourage or reward performance that is in alignment with the clinical innovation. See Krein et al. [11] for an example of this process. |
| **Develop and organize quality monitoring systems**  Develop and organize systems and procedures that monitor clinical processes and/or outcomes for the purpose of quality assurance and improvement.  ***Ancillary Material:***  This includes developing systems for monitoring through peer reviews, collecting data from patients and consumers, clinicians, and supervisors, and using administrative and electronic record data. This category of strategies also includes the design of disease-specific clinical registries, where clinical information and tools (graphical representations, real-time report cards, comparisons with benchmarks, etc.) are available to care team members. These systems may inform audit and feedback strategies. Some intensive fidelity monitoring activities (e.g., psychotherapy recordings) are more practical at random, but not infrequent, intervals. |
| **Develop disincentives**  Provide financial disincentives for failure to implement or use the clinical innovations.  ***Ancillary Material:***  In addition to direct financial disincentives, this strategy could include tying promotion decisions to the use of certain innovations. |
| **Develop educational materials**  Develop and format manuals, toolkits, and other supporting materials in ways that make it easier for stakeholders to learn about the innovation and for clinicians to learn how to deliver the clinical innovation.  ***Ancillary Material:***  Create eye-catching, easy-to-use educational documents. Distill complex information into easier-to-learn components. Consider teaching skills modularly. Use different forms of media, and target messages for different audiences. Educational materials should reflect principles of adult learning theory. Assessment of current, available technology infrastructure to accommodate educational media (e.g., firewalls, old hardware, old software) is merited. Consider how the educational materials will be used over time. For example, will the educational materials’ primary use be to train new or rotating staff; or to refresh staff knowledge; or to be incorporated into existing supervision, competency, and performance review structures. Educational materials may be refined through the use of formative evaluation feedback.  Relevant suggestions are provided via the REP framework, under its ‘packaging’ domain [7]. Further support related to developing educational materials can be found on the Training Within Industry Service website [29]. |
| **Develop resource sharing agreements**  Develop partnerships with organizations that have resources needed to implement the innovation.  ***Ancillary Material:***  For example, this could involve data sharing agreements, agreements to share necessary equipment (e.g., telemedicine equipment), or sharing the cost of bringing in experts who provide training and consultation. Resource sharing agreements could involve formal memorandums of understanding (MOUs), or be much more informal in nature. |
| **Distribute educational materials**  Distribute educational materials (including guidelines, manuals and toolkits) in person, by mail, and/or electronically.  ***Ancillary Material:***  None |
| **Facilitate relay of clinical data to providers**  Provide as close to real-time data as possible about key measures of process/outcomes using integrated modes/channels of communication in a way that promotes use of the targeted innovation.  ***Ancillary Material:***  For recommendations regarding how to introduce innovation or change of any kind into existing work flows, please see May [30]. |
| **Facilitation**  A process of interactive problem solving and support that occurs in a context of a recognized need for improvement and a supportive interpersonal relationship.  ***Ancillary Material:***  Facilitation can be internal or external to a system. This interactive support process can include a combination of any implementation strategies, and typically bundles multiple strategies as needed. |
| **Fund and contract for the clinical innovation**  Governments and other payers of services issue requests for proposals to deliver the innovation, use contracting processes to motivate providers to deliver the clinical innovation, and develop new funding formulas that make it more likely that providers will deliver the innovation.  ***Ancillary Material:***  None |
| **Identify and prepare champions**  Identify and prepare individuals who dedicate themselves to supporting, marketing, and driving through an implementation, overcoming indifference or resistance that the intervention may provoke in an organization.  ***Ancillary Material:***  This strategy includes preparing individuals for their role as champions. Champions are primarily internal to the organization. Additional issues raised include the need for guidance regarding:   1. Methods and considerations related to the selection and identification of champions. Social network theory and methods may be useful in this regard. 2. Training and or providing champions support materials. 3. Addressing incentives or disincentives to the champion role. 4. Whether there are needs for champions at different levels of an organization (e.g., clinic, region, national).   Champions are often distinguished from opinion leaders. Opinion leaders may be considered more of an objective third party with relevant expertise. |
| **Identify early adopters**  Identify early adopters at the local site to learn from their experiences with the practice innovation.  ***Ancillary Material:***  Early adopters are a good pool for identifying implementation champions. Recruit early adopters to attend stakeholder meetings to present their experiences. Investigating the adoption chasm between early adopters and the early majority has been found to be useful. Different engagement techniques for these two groups are typically needed. For further discussion see Moore [19]. |
| **Increase demand**  Attempt to influence the market for the clinical innovation to increase competition intensity and to increase the maturity of the market for the clinical innovation.  ***Ancillary Material:***  One way of increasing demand is to educate patients about the clinical innovation so that they demand it from their providers (e.g., what pharmaceutical companies do). |
| **Inform local opinion leaders**  Inform providers identified by colleagues as opinion leaders or ‘educationally influential’ about the clinical innovation in the hopes that they will influence colleagues to adopt it.  ***Ancillary Material:***  The opinions of individuals who refer people to services, or who initiate the connection to services also function in a key opinion role. Keeping opinion leaders informed from pre-implementation through maintenance of the clinical innovation is important. Ensuring that opinion leaders do not serve as implementation obstacles if they are not actively promoting the innovation is also important. |
| **Intervene with patients/consumers to enhance uptake and adherence**  Develop strategies with patients to encourage and problem solve around adherence.  ***Ancillary Material:***  This includes patient/consumer reminders and financial incentives to attend appointments. Feedback regarding patient/consumers' understanding and use of the treatment is also important to collect. |
| **Involve executive boards**  Involve existing governing structures (e.g., boards of directors, medical staff boards of governance) in the implementation effort, including the review of data on implementation processes.  ***Ancillary Material:***  Other types of leadership with ‘top-down’ powers may be involved for settings that do not have a governing board. Examples include administrative leadership, clinical leadership, policy makers, and insurance providers or other payment systems. |
| **Involve patients/consumers and family members**  Engage or include patients/consumers and families in the implementation effort.  ***Ancillary Material:***  Feedback from stakeholders can be obtained at any stage of the implementation process depending on the needs and goals of project. Involving stakeholders in the pre-implementation phase for many innovations is advantageous. Training in the innovation, and relevant advocacy, may also be included in stakeholder involvement. Informal caregivers such as neighbors, friends, and other key sources of support may also be prudent to include. |
| **Make billing easier**  Make it easier to bill for the clinical innovation.  ***Ancillary Material:***  Making billing easier might involve requiring less documentation, ‘block’ funding for delivering the innovation, and creating new billing codes for the innovation. Developing progress note templates to facilitate documentation of the clinical innovation can also decrease the burden for obtaining payment. |
| **Make training dynamic**  Vary the information delivery methods to cater to different learning styles work contexts, and shape the training in the innovation to be interactive.  ***Ancillary Material:***  Making training dynamic includes efforts to divide material into small time intervals, the use of small group breakouts, audience response systems, and other measures, such as having learners try new skills between training sessions. Interactive components of training can be very dynamic with participants actively contributing to the training content, engaging in problem solving, and identifying solutions that can be tested. |
| **Mandate change**  Have leadership declare the priority of the innovation and their determination to have it implemented.  ***Ancillary Material:***  It is important to ensure that the individuals mandating the change have the power to do so, as implementers often lack such authority. Working with organizational leadership to develop buy-in and lobby for a change mandate is often needed. It can also be important to inform other stakeholders (e.g., auditors, groups that review services for billing) about the mandate to ensure they are on the same page. |
| **Model and simulate change**  Model or simulate the change that will be implemented prior to implementation.  ***Ancillary Material:***  Computer simulations, walkthrough simulation exercises, or modeling the potential overall impact of stakeholder's behavior change may be used. System dynamics modeling is one example of a specific method that may be used [20]. This approach is often more relevant for complex multi-component innovations. |
| **Obtain and use patients/consumers and family feedback**  Develop strategies to increase patient/consumer and family feedback on the implementation effort.  ***Ancillary Material:***  This can continue throughout the implementation effort. Strategies could include complaint forms, or methods, which funnel feedback to change managers or advisory boards. Consider whether anonymous feedback formats are appropriate. |
| **Obtain formal commitments**  Obtain written commitments from key partners that state what they will do to implement the innovation.  ***Ancillary Material:***  Formal commitments should clarify roles, responsibilities, and detail tangible and non-tangible benefits (e.g., community partnerships). Ensure that key roles are supported within the organization (e.g., workload release credit for providing and receiving supervision in a new clinical practice). Formal commitments in no way diminish the importance of informal commitments to a change effort. |
| **Organize clinician implementation team meetings**  Develop and support teams of clinicians who are implementing the innovation and give them protected time to reflect on the implementation effort, share lessons learned, and support one another’s learning.  ***Ancillary Material:***  None |
| **Place innovation on fee for service lists/formularies**  Work to place the clinical innovation on lists of actions for which providers can be reimbursed (e.g., a drug is placed on a formulary, a procedure is now reimbursable).  ***Ancillary Material:***  None |
| **Prepare patients/consumers to be active participants**  Prepare patients/consumers to be active in their care, to ask questions, and specifically to inquire about care guidelines, the evidence behind clinical decisions, or about available evidence-supported treatments.  ***Ancillary Material:***  Preparing consumers to inquire about specific practices can involve asking questions, and educating patients/consumers about the existence of treatments supported by evidence, as well as explicitly inviting them into the process of treatment decision-making. |
| **Promote adaptability**  Identify the ways a clinical innovation can be tailored to meet local needs and clarify which elements of the innovation must be maintained to preserve fidelity.  ***Ancillary Material:***  Preserving fidelity to the innovation can be an uncertain process if the core elements of the innovation are not empirically defined. |
| **Promote network weaving**  Identify and build on existing high quality working relationships and networks within and outside the organization, organizational units, teams, etc. to promote information sharing, collaborative problem-solving, and a shared vision/goal related to implementing the innovation.  ***Ancillary Material:***  Individuals functioning as network weavers usually have external links outside of the community to bring in information and ideas. An example would be nurses and doctors who staff hospitals and skilled nursing facilities, and the patients who rotate among these facilities. Networks are somewhat more organic than collaboratives and are often enduring and durable. See: <http://www.networkweaver.com/> |
| **Provide clinical supervision**  Provide clinicians with ongoing supervision focusing on the innovation. Provide training for clinical supervisors who will supervise clinicians who provide the innovation.  ***Ancillary Material:***  Clearly defining the role of supervision and providing ongoing resources to ensure that it occurs can be important. Supervisor training often needs to include specific training in how to supervise the innovation.  See Nadeem et al. [13] for a discussion of the distinction between consultation and supervision. |
| **Provide local technical assistance**  Develop and use a system to deliver technical assistance focused on implementation issues using local personnel.  ***Ancillary Material:***  Local technical assistants can be connected with a broader or centralized network of technical assistants. Technical assistance for both the clinical innovation and the implementation processes may be important. For example, the VA aims to have mental health Evidence-Based Psychotherapy coordinators, Military Sexual Trauma coordinators, and OEF/OIF/OND coordinators in each facility who can provide technical assistance to other local clinicians for relevant initiatives. |
| **Provide ongoing consultation**  Provide ongoing consultation with one or more experts in the strategies used to support implementing the innovation.  ***Ancillary Material:***  Ongoing consultations could include in-person or distance consultation and feedback on taped clinical encounters. Consultations are tailored to the clinician’s actual practice, thus, differentiating a consultation from ongoing trainings. Feedback may be from a consultant external to the organization, which distinguishes consultation from clinical supervision. Some practice changes can involve a recertification process, thus, involving consultation ensures adequate fidelity. Consultation may also be necessary for non-clinical staff such as administrators and those responsible for billing, constructing feedback systems, or other staff with duties that impact the implementation process. |
| **Purposely reexamine the implementation**  Monitor progress and adjust clinical practices and implementation strategies to continuously improve the quality of care.  ***Ancillary Material:***  It is beneficial to use a concrete schedule for monitoring rather than ‘as needed.’ Time-sensitive benchmarks for determining when adjustments are needed have also been found to be useful. |
| **Recruit, designate, and train for leadership**  Recruit, designate, and train leaders for the change effort.  ***Ancillary Material:***  Change efforts require certain types of leaders, and organizations may need to recruit accordingly, rather than assuming that their current personnel can implement the change. Designated change leaders can include an executive sponsor and a day-to-day manager of the effort. Change leaders should consider how to establish effective supervisory lines for clinical practice innovations that are enacted by clinicians when the change leader does not have similar clinical responsibilities. |
| **Remind clinicians**  Develop reminder systems designed to help clinicians to recall information and/or prompt them to use the clinical innovation.  ***Ancillary Material:***  Reminders could be patient or encounter-specific, provided verbally, on paper, or electronically. Computer-aided decision support, and drug dosages are included in this strategy. Reminders may be delivered at various time points (prior to service, during service, or following service delivery). |
| **Revise professional roles**  Shift and revise roles among professionals who provide care, and redesign job characteristics.  ***Ancillary Material:***  Revising professional roles includes the expansion of roles to cover provision of the clinical innovation and the elimination of service barriers to care, including personnel policies. |
| **Shadow other experts**  Provide ways for key individuals to directly observe experienced people engage with or use the targeted practice change/innovation.  ***Ancillary Material:***  While shadowing traditionally has involved in-person observation, creative use of technology may provide additional opportunities for individuals to observe and learn from those experienced in the innovation. |
| **Stage implementation scale up**  Phase implementation efforts by starting with small pilots or demonstration projects and gradually moving to a system wide rollout.  ***Ancillary Material:***  This involves an iterative process that often results in adaptations. Strategies for integrating pilot feedback into the scale-up or spread process should be established in advanced. Depending on the innovation, piloting may also involve phasing in elements or components of the practice change. Many innovations involve more than one service (e.g., inpatient and outpatient; primary care and specialty care), and the scaling-up or spread process may have different needs to address the interactions among services (e.g. needs related to ensure continuity of care while connecting services). For more details see the Institute for Healthcare Improvement’s white paper, which describes a framework for spread [12]. |
| **Start a dissemination organization**  Identify or start a separate organization that is responsible for disseminating the clinical innovation. It could be a for-profit or non-profit organization.  ***Ancillary Material:***  This strategy can address concerns (e.g., conflict of interest) for situations in which it is desirable to have fidelity monitors that are independent from the care setting. The dissemination organization could be a for-profit or nonprofit organization. The organization could be ‘licensed’ by a university, if the innovation was born within an academic setting. It is important for dissemination organizations to be aware of organizations' approaches to implementing other interventions in order to build upon existing practices. |
| **Tailor strategies**  Tailor the implementation strategies to address barriers and leverage facilitators that were identified through earlier data collection.  ***Ancillary Material:***  The tailoring process tends to be idiosyncratic and driven by multiple factors. It is important to identify the core components of the intervention and implementation strategies that are required to maintain fidelity and effectiveness, and to distinguish those components amenable to modification/adaptation. Flottorp et al. [14] and Langley et al. [15] provide guidance regarding many of the multiple factors to consider. Wensing et al. [16, 17] provide an example of a structured approach to tailoring strategies. |
| **Use advisory boards and workgroups**  Create and engage a formal group of multiple kinds of stakeholders to provide input and advice on implementation efforts and to elicit recommendations for improvements.  ***Ancillary Material:***  Consider how group composition (or heterogeneity) impacts stakeholder participation and take active steps to reduce response bias. For example, inclusion of supervisors and supervisees in these groups can be problematic and it may be a desirable strategy to ensure that these situations are avoided due to the power difference in the relationship. Supervisees, for example, may feel pressure to report positively to put a good face on for the supervisors, and supervisors may feel pressure to deny having any problems with implementation to save face. It can be useful to distinguish between internal stakeholders and representatives (in a participatory approach to maintain buy-in and relevance) versus external experts and advisors. Similarly, depending on the input or oversight need, the workgroup composition may include multiple-level or multi-disciplinary stakeholders. |
| **Use an implementation advisor**  Seek guidance from experts in implementation.  ***Ancillary Material:***  This could include consultation with outside experts such as university-affiliated faculty members, or hiring quality improvement experts or implementation professionals. |
| **Use capitated payments**  Pay providers or care systems a set amount per patient/consumer for delivering clinical care.  ***Ancillary Material:***  This is an implementation strategy to the degree that it frees the clinician to provide services that they may have been disincentivized to provide under a fee-for-service structure. This may be helpful to motivate clinicians to use certain clinical innovations. These changes often come about as part of policy changes that alter fee structures, alter coverage, or add items to reimbursement formularies. |
| **Use data experts**  Involve, hire, and/or consult experts to inform management on the use of data generated by implementation efforts.  ***Ancillary Material:***  Consider engaging data experts early in the implementation planning process. |
| **Use data warehousing techniques**  Integrate clinical records across facilities and organizations to facilitate implementation across systems.  ***Ancillary Material:***  Records that include variables that can serve as outcome measures are particularly useful. When outcomes of interest are not available, it may be useful to examine proxy measures. |
| **Use mass media**  Use media to reach large numbers of people to spread the word about the clinical innovation.  ***Ancillary Material:***  Mass media may include television, newspapers, magazines, radio, electronic social media, listservs, mass email campaigns, mass mailings, and robocalls as methods for spreading information. Targets of these media campaigns may be clinicians, potential consumers of the innovation, or their associates. Other commonly used terms include marketing or social marketing. |
| **Use other payment schemes**  Introduce payment approaches (in a catch-all category).  ***Ancillary Material:***  Payment scheme approaches may involve prepayment and prospective payment for service, provider salaried service, the alignment of payment rates with the attainment of patient/consumer outcomes, and the removal or alteration of billing limits, such as numbers of encounters that are reimbursable. Payment may also be based on measures of treatment fidelity. Payment schemes are implementation strategies to the degree that they free the clinician’s time to provide the clinical innovation. Others strategies motivate clinicians to provide better service. |
| **Use train-the-trainer strategies**  Train designated clinicians or organizations to train others in the clinical innovation.  ***Ancillary Material:***  Restrictions regarding who can serve as a trainer are idiosyncratic to the innovation or practice change, for example, some innovations may require that supervisors have specific levels of education, training, or experience, and such restrictions should be explored in the planning phase. Train-the-trainer strategies may also apply to those responsible for administrative procedures, and who are part of implementing the innovation. |
| **Visit other sites**  Visit sites where a similar implementation effort has been considered successful.  ***Ancillary Material:***  Clarifying the goals of the site visit prior to making the visit is particularly useful. Comparing and contrasting the features of one’s own site with the comparison site in preparation for the visit may better inform the visit objectives. Clarifying goals, in part includes developing a plan for using the information upon returning to your setting. Identify adaptations made in implementing the innovation and any perceived impact on the effectiveness of the innovation/practice change. It is important to document facilitators and lessons learned. Much can be learned from visiting sites that have a strong track record for successfully implementing a wide variety of other innovations/practice changes. Consulting with sites where implementation has stalled or failed can also provide useful information. Sites also benefit from sharing implementation planning and execution notes virtually (i.e., information exchange is not limited to physical visits). |
| **Work with educational institutions**  Encourage educational institutions to train clinicians in the innovation.  ***Ancillary Material:***  This strategy fits well with innovations requiring clinical training and other skills where training expertise is more likely to be housed in educational institutions. |

Note. See Powell et al. [31] for additional references associated with strategies in the original compilation.

**References**

1. Helfrich CD, Li Y-F-. F, Sharp ND, Sales AE: Organizational readiness to change assessment (ORCA): Development of an instrument based on the promoting action on research in health services (PARIHS) framework. *Implement Sci* 2009, 4.

2. Lehman WEK, Greener JM, Simpson DD: Assessing organizational readiness for change. *J Subst Abuse Treat* 2002, 22:197–209.

3. Weiner BJ, Amick H, Lee S-YD-. YD: Conceptualization and measurement of organizational readiness for change: A review of the literature in health services research and other fields. *Med Care Res Rev* 2008, 65:379–436.

4. Deming WE: *Out of the Crisis*. Cambridge, MA: MIT Press; 1986.

5. Linderman K, Schroeder RG, Zaheer S, Choo AS: Six sigma: A goal-theoretic perspective. *J Oper Manag* 2003, 21:193–203.

6. Neumann MS, Sogolow ED: Replicating effective programs: HIV/AIDS prevention technology transfer. *AIDS Educ Prev* 2000, 12(5 Suppl):35–48.

7. Kilbourne AM, Neumann MS, Pincus HA, Bauer MS, Stall R: Implementing evidence-based interventions in health care: Application of the replicating effective programs framework. *Implement Sci* 2007, 2:1–10.

8. Pyne JM, Fortney JC, Curran GM, Tripathi S, Atkinson JH, Kilbourne AM, Hagedorn HJ, Rimland D, Rodriguez-Barradas MC, Monson T, Bottonari KA, Asch SM, Gifford AL: Effectiveness of collaborative care for depression in human immunodeficiency virus clinics. *Arch Intern Med* 2010, 171:23–31.

9. Curran GM, Mukherjee S, Allee E, Owen RR: A process for developing an implementation intervention: QUERI series. *Implement Sci* 2008, 3:1–11.

10. Hunt JB, Curran G, Kramer T, Mouden S, Ward-Jones S, Owen R, Fortney J: Partnership for implementation of evidence-based mental health practices in rural federally qualified health centers: Theory and methods. *Prog Community Health Partnersh* 2012, 6:389–398.

11. Krein SL, Bernstein SJ, Fletcher CE, Makki F, Godzweig CL, Watts B, Vijan S, Hayward RA: Improving eye care for veterans with diabetes: An example of using the QUERI steps to move from evidence to implementation: QUERI series. *Implement Sci* 2014, 3:1–11.

12. Massoud MR, Nielsen GA, Nolan K, Nolan T, Schall MW, Sevin CA: *A Framework for Spread: From Local Improvements to System-Wide Change*. Cambridge, Massachusetts: Institute for Healthcare Improvement; 2006.

13. Nadeem E, Gleacher A, Beidas RS: Consultation as an implementation strategy for evidence-based practices across multiple contexts: Unpacking the black box. *Adm Policy Ment Health* 2013, 40:439–450.

14. Flottorp SA, Oxman AD, Krause J, Musila NR, Wensing M, Godycki-Cwirko M, Baker R, Eccles MP: A checklist for identifying determinants of practice: A systematic review and synthesis of frameworks and taxonomies of factors that prevent or enable improvements in healthcare professional practice. *Implement Sci* 2013, 8:1–11.

15. Langley GJ, Moen R, Nolan KM, Nolan TW, Norman CL, Provost LP: *The Improvement Guide: A Practical Approach to Enhancing Organizational Performance*. 2nd edition. San Francisco, CA: Jossey-Bass, Inc.; 2009.

16. Wensing M, Huntink E, van Lieshout J, Godycki-Cwirko M, Kowalczyk A, Jäger C, Steinhäuser J, Aakhus E, Flottorp S, Eccles M, Baker R: Tailoring implementation of evidence-based practice for patients with chronic diseases. *PLOS One* 2014, 9:1–8.

17. Wensing M, Oxman A, Baker R, Godycki-Cwirko M, Flottorp S, Szecsenyi J, Grimshaw J, Eccles M: Tailored implementation for chronic diseases (TICD): A project protocol. *Implement Sci* 2011, 6:1–8.

18. Patton MQ: *Essentials of Utilization-Focused Evaluation*. Thousand Oaks, CA: Sage Publications, Inc.; 2012.

19. Moore GA: *Crossing the Chasm: Marketing and Selling Disruptive Products to Mainstream Customers*. 3rd edition. New York: Harper Business; 2014.

20. Homer JB, Hirsch GB: System dynamics modeling for public health: Background and opportunities. *Am J Public Health* 2006, 96:452–458.

21. Fischer MA, Avorn J: Academic detailing can play a key role in assessing and implementing comparative effectiveness research findings. *Health Aff (Millwood)* 2012, 31:2206–2212.

22. Soumerai SB, Avorn J: Principles of educational outreach (’academic detailing’) to improve clinical decision making. *JAMA* 1990, 4:549–556.

23. Positive deviance initiative: Discovery and action dialogue (DAD) cheat sheet [http://www.positivedeviance.org/pdf/DAD%20cheat%20sheet%202.pdf]

24. Pascale R, Sternin J, Sternin M: *The Power of Positive Deviance: How Unlikely Innovators Solve the World’s Toughest Problems*. Boston, MA: Harvard Business Press; 2010.

25. Bradley EH, Curry LA, Ramanadhan S, Rowe L, Nembhard IM, Krumholz HM: Research in action: Using positive deviance to improve quality of health care. *Implement Sci* 2009, 4:1–11.

26. Health Resources and Services Administration: Learning collaboratives [http://www.mchb.hrsa.gov/programs/learningcollaboratives/index.html]

27. Institute for Healthcare Improvement: Collaboratives [http://www.ihi.org/Engage/collaboratives/Pages/default.aspx]

28. Institute for Healthcare Improvement: *The Breakthrough Series: IHI’s Colaborative Model for Achieving Breakthrough Improvement*. Cambridge, Massachusetts: Institute for Healthcare Improvement; 2003.

29. Training Within Industry Service [http://www.trainingwithinindustry.net]

30. May C: Towards a general theory of implementation. *Implement Sci* 2013, 8:1–14.

31. Powell BJ, McMillen JC, Proctor EK, Carpenter CR, Griffey RT, Bunger AC, Glass JE, York JL: A compilation of strategies for implementing clinical innovations in health and mental health. *Med Care Res Rev* 2012, 69:123–157.
